# The efficacy and safety of combining different commercial Chinese polyherbal preparations with conventional Western drugs in the treatment of diabetic osteoporosis: a network meta-analysis

**DOI:** 10.3389/fphar.2026.1880450

**Published:** 2026-10-21

**Authors:** Xiaoting Ni, Yingting Guan, Shenghao Zhi, Xu Wang

**Affiliations:** 1 The First Clinical Medical College, Nanjing University of Chinese Medicine, Nanjing, China; 2 Jiangsu Province Hospital of Chinese Medicine, Nanjing, China

**Keywords:** commercial Chinese polyherbal preparation, diabetic osteoporosis, network meta-analysis, randomized controlled trial, systematic review

## Abstract

**Background:**

Biomedicine for diabetic osteoporosis (DOP) is often associated with serious side effects, such as osteonecrosis of the jaw and atypical femoral fractures. In contrast, recent clinical studies have highlighted the potential of commercial Chinese polyherbal preparations (CCPPs) as effective alternatives. These studies suggest that CCPPs help increase bone mineral density, relieve pain, regulate bone turnover markers, and lower blood glucose, thereby improving osteoporosis. This study aimed to evaluate the effectiveness of six CCPPs combined with biomedicine for the treatment of DOP. A network meta-analysis (NMA) was used to compare and rank the therapeutic effects of these CCPPs.

**Materials and Methods:**

We conducted a comprehensive search of multiple databases up to November 2025 to screen for studies assessing the effectiveness of CCPPs for DOP. Two independent reviewers performed study screening, data extraction, and risk of bias assessment. Risk of bias was evaluated using the Cochrane Risk of Bias tool version 2.0, and the certainty of evidence was graded according to the Confidence in Network Meta-Analysis (CINeMA) framework. Continuous outcomes were expressed as mean differences (MD) with 95% confidence intervals (CI); dichotomous outcomes were summarized as odds ratios (OR) or risk ratios (RR) with their corresponding 95% CIs. All analyses were completed using StataMP 18 software, and treatment rankings were determined using the surface under the cumulative ranking curve (SUCRA) method.

**Results:**

This study included 34 studies involving 3,720 patients with DOP, evaluating six different CCPP regimens. All CCPP regimens showed benefits in increasing bone mineral density, relieving pain, regulating bone turnover markers, and lowering blood glucose. Compared with biomedicine alone, CCPPs combined with biomedicine demonstrated superior outcomes in overall response rate, bone mineral density, bone turnover markers, alkaline phosphatase reduction, and fasting blood glucose, while also showing a lower incidence of adverse events. Specifically, the Guskang (GSK) regimen ranked highest in overall response rate; Xianling Gubao (XLGB), Jintiange (JTG), and GSK showed notable effects on improving bone mineral density; JTG was best for increasing osteocalcin levels; XLGB ranked highest for calcium and phosphorus; and Liuwei Dihuang Wan (LWDHW) produced the greatest reduction in fasting blood glucose. GL had the lowest incidence of adverse events.

**Conclusion:**

Compared with biomedicine alone, combining CCPPs with biomedicine for DOP treatment improves clinical outcomes. However, high-quality, rigorously designed clinical trials are needed to validate these preliminary results.

**Systematic Review Registration:**

https://www.crd.york.ac.uk/PROSPERO/view/CRD420251240840, identifier CRD420251240840.

## 1 Introduction

Diabetes is a major global public health challenge characterized by chronic hyperglycemia, metabolic disturbances, multisystem impairment, and numerous complications. Population aging and changes in lifestyle and dietary patterns have further increased the global burden of diabetes. According to epidemiological statistics, the number of individuals affected by diabetes is projected to reach 853 million by 2050 (Álvarez-Cubela et al., 2025). This condition often gives rise to a secondary metabolic bone disorder known as diabetic osteoporosis, which is defined by reduced bone density, deterioration of bone tissue structure, heightened bone brittleness, and a substantially elevated risk of fractures (Cao et al., 2025). Patients with diabetes have a higher risk of osteoporosis and fragility fractures than the general population. Osteoporosis is particularly prevalent in type 1 diabetes, affecting approximately 90.1% of patients (Dutta et al., 2016). In contrast, type 2 diabetes patients exhibit a 37.8% prevalence rate, with women more likely to be affected (44.8% versus 37.0%) (Si et al., 2020). Globally, over 90,000 osteoporotic fractures annually are linked to this disorder, representing a substantial danger to public health and economic progress (Johnell and Kanis, 2006). The development of diabetic osteoporosis arises from intricate pathological processes, such as the buildup of advanced glycation end products, persistent high blood sugar levels, and oxidative stress, all of which damage collagen cross-linking and weaken bone structure (Vashishth, 2007). Current evidence further suggests that impaired bone remodeling, reduced osteoblast function, and deterioration of bone material quality contribute to skeletal fragility in diabetes, even when bone mineral density is not markedly reduced (Khosla et al., 2021). Individuals with diabetes also face significantly higher rates of post fracture disability and mortality compared to non diabetic counterparts, emphasizing the critical need for improved preventive and therapeutic approaches (Center et al., 2007).

Currently, modern medical treatment for diabetic osteoporosis mainly adopts a comprehensive strategy combining glucose-lowering drugs with anti-osteoporosis medications, such as bisphosphonates, teriparatide, and denosumab (Ferrari et al., 2018). These therapies primarily target glycemic control, bone resorption, or bone formation and remain central to current clinical management. However, long term use of these drugs may be associated with potential adverse effects including osteonecrosis of the jaw, atypical femoral fractures, cardiovascular risk, and neuropsychiatric symptoms (Oteo-Álvaro et al., 2023; Rubin et al., 2020). Moreover, some glucose lowering drugs, particularly thiazolidinediones, may exacerbate bone loss through activation of peroxisome proliferator activated receptor γ (PPAR γ), complicating the balance between glycemic control and skeletal health (Kahn et al., 2008). Therefore, the simultaneous management of glucose metabolism and skeletal fragility remains a clinical challenge, supporting the evaluation of complementary therapeutic strategies with acceptable safety profiles.

Commercial Chinese polyherbal preparations (CCPPs) are standardized formulations developed according to traditional Chinese medicine theory and prepared in defined dosage forms for clinical use. Experimental studies suggest that constituents such as Epimedium and Drynaria exert multiple target effects on bone metabolism (Song et al., 2025). Their underlying mechanisms involve enhancing the differentiation of bone-forming cells, suppressing the activity of bone-resorbing cells, and improving the blood supply to bone tissues via the OPG/RANKL/RANK signaling pathway (Chen et al., 2021). Several randomized controlled trials have shown that CCPPs, such as Qianggu capsule and Xianling Gubao capsule, combined with standard treatment may improve bone mineral density, relieve pain, and regulate bone metabolism markers compared with standard treatment alone (Wang K. et al., 2024; Liang et al., 2022). Animal studies also provide mechanistic support. For example, Xianling Gubao capsule may promote osteoblast differentiation by regulating the miR 100 5p, KDM6B, and RUNX2 pathway (Ai et al., 2021).

Despite the large number of clinical trials, the comparative ranking of these interventions remains unclear. Most existing systematic reviews rely on pairwise meta analysis and primarily compare individual CCPPs with placebo or standard care. Consequently, the relative effectiveness of different CCPP regimens remains uncertain (Man et al., 2025). Network meta analysis can synthesize direct and indirect evidence and simultaneously compare multiple CCPP regimens. Moreover, the relative effects of different CCPPs across bone mineral density, bone turnover markers, glycemic outcomes, clinical response, and safety have not been comprehensively compared within a unified analytical framework. In contrast, network meta analysis offers a more comprehensive approach by synthesizing both direct and indirect data when direct comparisons are unavailable, allowing multiple CCPP regimens to be compared and ranked simultaneously.

Therefore, this study aimed to compare the relative efficacy and safety of different oral CCPPs combined with conventional Western medical treatment for diabetic osteoporosis using a network meta analysis. Key outcomes included bone mineral density, bone turnover markers, blood glucose, clinical response, and adverse events. This approach was intended to provide comparative evidence for clinical decision making and future research.

## 2 Methods

This network meta-analysis was conducted following the PRISMA extension guidelines for systematic reviews, particularly those related to network meta-analyses of healthcare interventions. The review protocol was registered with the Prospective Register of International Systematic Reviews (PROSPERO: CRD420251240840).

### 2.1 Inclusion and exclusion criteria

#### 2.1.1 PICOS criteria

##### 2.1.1.1 Participants

Patients with type 2 diabetes mellitus diagnosed according to World Health Organization or relevant national guidelines, and concurrently diagnosed with osteoporosis by dual-energy X-ray absorptiometry (T-score ≤ −2.5 at the lumbar spine or hip). No restrictions were applied regarding sex, cause, ethnicity, or severity.

##### 2.1.1.2 Interventions

Any oral CCPP combined with biomedicine. The specific CCPPs included Xianling Gubao capsule, Jintiange capsule, Tangmaikang granules, Gushukang capsule/granules, Liuwei Dihuang Wan, and Guli capsule.

##### 2.1.1.3 Comparators

Conventional biomedicine referred to the conventional background management of osteoporosis and/or type 2 diabetes reported in the original trials. Depending on the study, biomedicine included a low salt, low fat diabetes diet, calcium and vitamin D supplementation, bisphosphonates such as alendronate or zoledronic acid, active vitamin D analogues such as calcitriol or alfacalcidol, insulin, oral glucose lowering agents, or combinations of these therapies. The specific composition, dose, frequency, and duration of biomedicine were not restricted and varied across studies. For studies with complex treatment regimens, intervention and comparator groups were classified according to the original randomized design, and CCPP components were distinguished from background Western medical therapies. Trial specific treatment regimens are presented in Supplementary Appendix Table S2.1.

##### 2.1.1.4 Outcomes

Dichotomous variables included total effective rate (TER) and incidence of adverse events. TER was defined as the proportion of participants classified as responders according to the criteria reported in each original trial. The criteria varied across studies and generally incorporated improvements in bone mineral density, pain or VAS scores, TCM syndrome scores, or clinical symptoms. Because no uniform definition of treatment response was applied across trials, TER was extracted according to the definition used in each study. Continuous variables included at least one of the following: lumbar spine bone mineral density, femoral neck bone mineral density, femoral trochanter bone mineral density, Ward’s triangle bone mineral density, osteocalcin, alkaline phosphatase, calcium, phosphorus, fasting blood glucose, or VAS score. BMD was considered a surrogate measure of skeletal efficacy, whereas osteocalcin and alkaline phosphatase were interpreted as secondary pharmacodynamic outcomes. Incident fractures, including new vertebral fractures, trabecular bone score, and other bone microstructural parameters were not available for quantitative synthesis in the eligible trials.

Study types included randomized controlled trials (RCTs) that were either released within China or abroad, with no consideration given to how the participants were assigned or whether the researchers masked the participants to the treatment, These studies all included comprehensive patient data, with no limitations imposed based on language requirements.

#### 2.1.2 Exclusion criteria

1. The randomized comparison did not meet the predefined intervention framework of CCPPs combined with biomedicine versus biomedicine alone, including studies in which CCPPs were used without appropriate background Western medical treatment or where CCPP -containing regimens were assigned to the comparator group.
2. The treatment group received additional non- CCPP regimens (e.g., CCPPs combined with acupuncture, health exercises, or decoctions, hospital preparations, etc.) beyond the predefined interventions.
3. The study had no extractable outcome data.

### 2.2 Literature search strategy

Two researchers systematically searched PubMed, Embase, Cochrane Library, Web of Science, China National Knowledge Infrastructure (CNKI), Wanfang Data, VIP database, and Chinese Biomedical Literature Database for studies on the effectiveness and safety of CCPPs for DOP, covering publications up to November 2025. We also reviewed conference proceedings, reference lists, and consulted experts. The search followed the PICOS framework and included keywords such as “diabetic osteoporosis,” “Chinese patent medicine,” and “randomized controlled trial.” Both MeSH terms and free text were used, with a unified strategy for both Chinese and English databases. The detailed search strategy is provided in Supplementary Appendix S1.

### 2.3 Literature screening and data extraction

Two independent researchers identified studies through literature searches and managed references using EndNote 20 software. After removing duplicates via software and manual checks, initial screening was performed based on titles and abstracts; studies that did not meet inclusion criteria were excluded. Full texts were then further assessed according to the prespecified inclusion and exclusion criteria. Disagreements between researchers were resolved through discussion, with consultation of a third arbitrator when necessary. A standard predesigned form was used to extract data, including: (1) basic information (first author, publication year); (2) participant characteristics (sample size, age, sex, BMI, disease duration, and baseline BMD where reported); (3) intervention details (CCPP name, composition, dosage, treatment duration, and comparator regimen, including the type, dose, frequency, and duration of biomedicine); (4) outcome data (means and standard deviations at each time point, number of events); and (5) information required for risk of bias assessment. For trials with complex intervention descriptions, treatment arms were verified against the original publication to ensure accurate classification of CCPPs and biomedicine components.

### 2.4 Risk of bias assessment

All selected studies were evaluated for potential biases using the Cochrane Collaboration’s tool for assessing risk of bias in randomized trials (RoB 2.0) (Sterne et al., 2019). Evaluations of bias across five critical areas were conducted by two separate reviewers: (1) the method of random sequence generation, (2) deviations from the planned interventions, (3) incomplete data reporting, (4) the manner in which outcomes were measured, and (5) the selection process for reported results. Differences of opinion were addressed by mutual consultation, and a third reviewer was brought in to assist whenever consensus could not be reached. For each research area, an evaluation was conducted in accordance with the criteria outlined in RoB 2.0, and the risk of bias was categorized as “low risk,” “some concerns,” or “high risk” in accordance with the RoB 2 framework. Subsequently, an integrated assessment of risk for each investigation was conducted. Studies with all domains judged as low risk were classified as low risk. Studies with one or more domains rated as “some concerns” were generally classified as having some concerns unless the accumulated concerns substantially affected confidence in the results. Studies with one or more high-risk domains or multiple important concerns that substantially reduced confidence in the findings were classified as high risk. Additional specifics can be found in Supplementary Appendix S4.

### 2.5 Statistical analysis

Data integration and visualization were performed using Stata MP 18 statistical software. The study adopted a frequentist statistical framework and used maximum likelihood estimation to calculate treatment effects, with standard errors (SE) used to assess the precision of parameter estimates. For different outcome types, dichotomous variables were expressed as risk ratios (RR) and odds ratios (OR) with 95% confidence intervals (CI), while continuous variables were expressed as mean differences (MD) with 95% CI. The direction of MD estimates was interpreted according to the clinical meaning of each outcome and the reference treatment in each comparison. For outcomes where higher values indicate improvement, positive MD values represented favorable effects, whereas for outcomes where lower values indicate improvement, negative MD values represented favorable effects. Heterogeneity was assessed using the τ^2^ statistic, with classification criteria: low heterogeneity (τ^2^ < 0.04); low to moderate heterogeneity (0.04 ≤ τ^2^ < 0.16); moderate to high heterogeneity (0.16 ≤ τ^2^ < 0.36); high heterogeneity (τ^2^ ≥ 0.36) (Turner et al., 2012; da Costa and Juni, 2014). The plausibility of the transitivity assumption was assessed qualitatively by comparing clinically relevant potential effect modifiers across treatment comparisons, including age, sex distribution, diabetes duration, baseline disease severity as reflected by baseline BMD where available, treatment duration, and the type and composition of background biomedicine. The assessment considered the degree of overlap and potential imbalance in these characteristics across treatment comparisons. Because several variables, particularly baseline BMD and some participant characteristics, were incompletely reported, transitivity was assessed using the available study level data and was not considered definitively established. Comparisons between treatment regimens used multiple treatment comparison models, and network graphs visualized the relationships among different interventions. Treatment rankings were summarized using surface under the cumulative ranking curve (SUCRA) values ranging from 0 to 1, with higher values indicating a higher relative treatment ranking. SUCRA rankings were interpreted together with effect estimates, 95% CIs, and the certainty of evidence assessed by CINeMA, and were considered exploratory rather than definitive evidence of treatment superiority. To assess potential publication bias, funnel plots were visually inspected. The robustness of the findings was considered together with risk of bias assessment and CINeMA certainty evaluation. Sensitivity analyses excluding studies with high risk of bias were not performed because removing these studies would substantially reduce the available evidence network for several comparisons and potentially compromise the stability of the network estimates.

### 2.6 Quality assessment

The certainty of evidence from the network meta-analysis was assessed using the Confidence in Network Meta-Analysis (CINeMA) framework. CINeMA evaluates the certainty of evidence across six domains: within-study bias, reporting bias, indirectness, imprecision, heterogeneity, and inconsistency. Based on these domains, an overall confidence rating was assigned to each network estimate (Nikolakopoulou et al., 2020; Papakonstantinou et al., 2020).

## 3 Results

### 3.1 Literature search results

The initial search yielded 1,530 records. After deduplication using EndNote, 505 records remained. After title and abstract screening, 359 records were excluded for not meeting the inclusion criteria. A total of 146 studies underwent full-text assessment, of which 112 were excluded (16 due to no relevant outcome, 75 due to irrelevant interventions, 4 due to ineligible population, 11 due to incomplete data, 6 due to outcome mismatch). Ultimately, 34 studies (S1-S34) were included in the meta-analysis (Figure 1).

**FIGURE 1:**
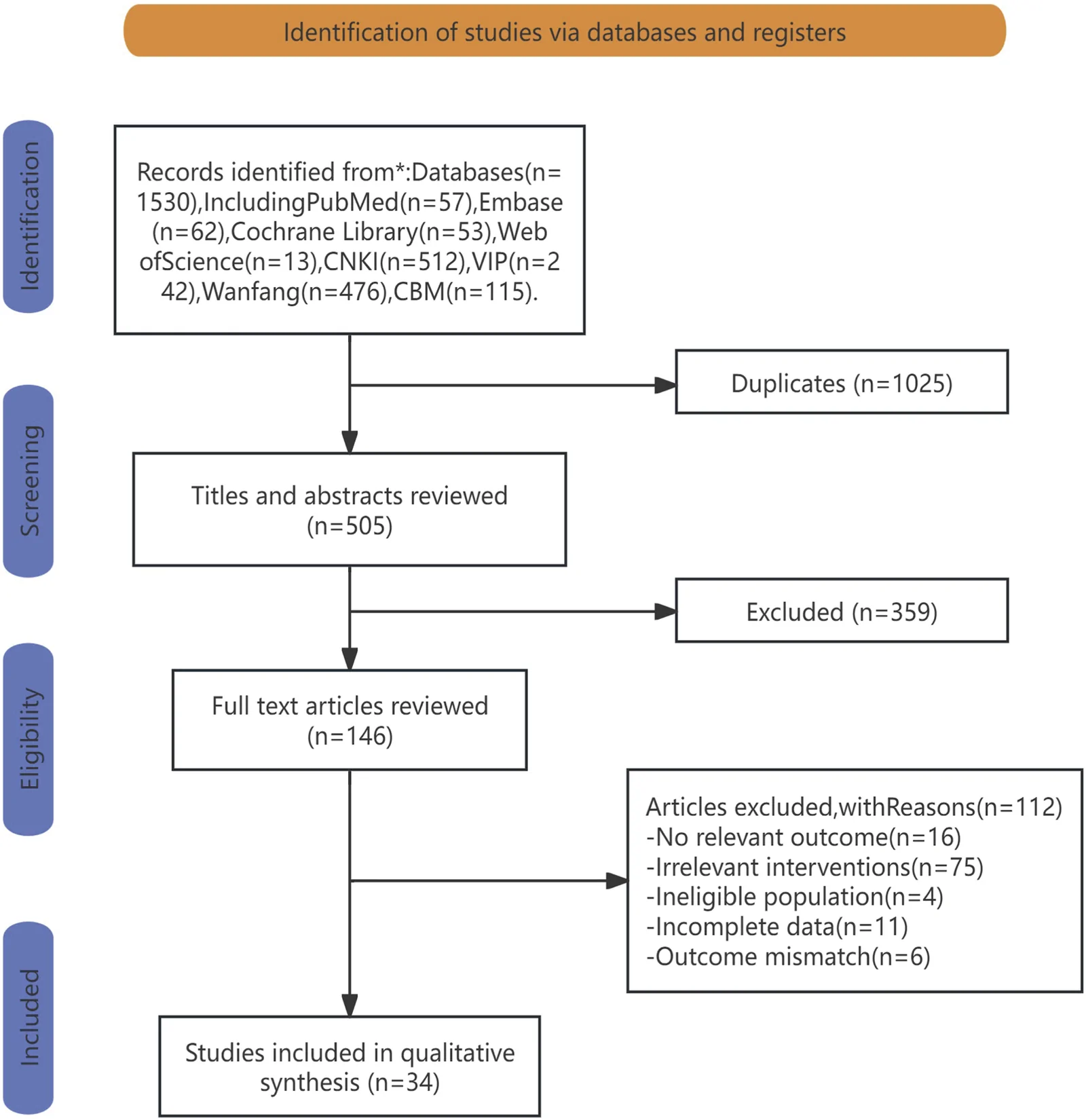
Flow chart of literature screening.

### 3.2 Characteristics of included studies

This NMA included 34 RCTs (S1-S34) published in Chinese between 2008 and 2025, involving a total of 3,720 participants. The control group received biomedicine alone, while the treatment group received biomedicine combined with CCPPs, including Xianling Gubao capsule, Jintiange capsule, Tangmaikang granules, Gushukang capsule/granules, Liuwei Dihuang Wan, and Guli capsule. The composition of biomedicine varied across studies and mainly included bisphosphonates such as alendronate or zoledronic acid, active vitamin D analogues such as calcitriol or alfacalcidol, calcium and vitamin D supplementation, and glucose lowering therapy when prescribed. The specific regimen, dose, frequency, and treatment duration differed among trials, reflecting clinical heterogeneity in the comparator treatment. The mean age of patients ranged from 50 to 73 years, but five RCTs did not specify age range. Sample sizes ranged from 48 to 300 participants. Treatment duration ranged from 1 month to 1 year. Patient characteristics showed some overlap across treatment comparisons, although age, sex distribution, and diabetes duration varied among studies and were incompletely reported in several trials. All participants met the eligibility criteria for type 2 diabetes mellitus with osteoporosis, but baseline BMD was not consistently reported, limiting detailed comparison of baseline disease severity across treatment comparisons. Potential effect modifiers considered in the assessment of transitivity included age, sex distribution, diabetes duration, baseline BMD where available, treatment duration, and background biomedicine. Variation was more evident in treatment duration and background biomedicine, whereas the available patient characteristics were broadly comparable but not uniformly distributed across treatment comparisons. Trial specific treatment details and corresponding study IDs (S1-S34) are provided in Supplementary Appendix Table S2.1.

Six CCPPs were tested across the 34 trials. The selection of CCPPs varied across studies, but commonly used herbs included Epimedium (Yinyanghuo), Salvia miltiorrhiza (Danshen), Prepared Rehmannia Root (Shudihuang), Psoralea corylifolia (Buguzhi), Astragalus membranaceus (Huangqi), and Codonopsis pilosula (Dangshen). These herbs are known for their effects of nourishing yin and tonifying the kidney, activating blood circulation and removing stasis, and supplementing qi and strengthening the spleen, which are especially applicable to symptoms of diabetic osteoporosis such as back pain, knee weakness, and lower limb weakness. Plant names were verified through the Real World Flora Online or MPNS. Further details on the TCM effects, ingredients, chemical constituents, and pharmacological actions of these herbs are provided in Supplementary Appendix Table S2.2.

### 3.3 Risk of bias, certainty of evidence, and consistency

The distribution of risk of bias across included studies is summarized as follows (detailed data in Supplementary Appendix S4). In the domains of outcome data completeness, outcome measurement, and selective reporting, all included studies were assessed as having low risk of bias. However, in the randomization process domain, nine studies mentioned randomization but did not clearly describe methods for allocation concealment or blinding, leading to a classification of “some concerns.” Regarding adherence to interventions, all studies were assessed as having “some concerns.” Overall, nine studies (26.5%) had high risk of bias, and 25 studies (73.5%) had some concerns. Consistency was assessed by testing direct and indirect evidence, and most comparisons showed no significant statistical heterogeneity (P > 0.05). τ^2^ analysis indicated low-to-moderate overall network heterogeneity. Using CINeMA to assess evidence quality, most pairwise comparisons had low-to-moderate confidence, and no evidence showed high confidence (Supplementary Appendix S8). The plausibility of transitivity was assessed by comparing the available distributions of age, sex, diabetes duration, baseline BMD, treatment duration, and background biomedicine across treatment comparisons. Patient characteristics showed broad clinical overlap, although some variation in age, sex distribution, and diabetes duration was observed and several characteristics were incompletely reported. Treatment duration ranged from 1 month to 1 year, and background biomedicine varied across studies, including bisphosphonates, active vitamin D analogues, calcium and vitamin D supplementation, and glucose lowering therapy. Baseline BMD was not consistently reported and therefore could not be systematically compared across all treatment comparisons. Based on the available study level data, transitivity was considered broadly plausible, although residual uncertainty could not be excluded. Publication bias assessment: funnel plot symmetry testing did not show significant bias (Supplementary Appendix S9). Considering the methodological limitations of the included studies, the potential influence of high risk of bias studies was carefully considered. However, because some treatment comparisons were supported by a limited number of trials, excluding all studies with high risk of bias may lead to insufficient network connectivity and unstable estimates. Therefore, the interpretation of comparative effects was based on the combined consideration of risk of bias assessment, CINeMA certainty ratings, and effect estimates.

### 3.4 Primary outcomes: network meta-analysis results

#### 3.4.1 Lumbar spine bone mineral density

The NMA for lumbar spine BMD included 22 trials involving 2,521 participants. Six CCPP regimens combined with biomedicine were available for indirect comparisons (Figure 2A). As shown in the forest plot (Figure 3A), compared with biomedicine alone, five CCPP regimens significantly increased lumbar spine BMD. Regimens involving GSK, TMK, JTG, XLGB, and GL showed statistically significant improvements. According to SUCRA rankings, the top three interventions were: GL + biomedicine [MD = 0.18, 95% CI (0.09, 0.27), SUCRA = 97.6%; moderate confidence of evidence], XLGB + biomedicine [MD = 0.11, 95% CI (0.08, 0.15), SUCRA = 80.7%; moderate confidence of evidence], and LWDHW + biomedicine [MD = 0.08, 95% CI (0.02, 0.14), SUCRA = 54.6%; moderate confidence of evidence]. The direction of MD estimates was interpreted according to the reference treatment in each comparison. After considering the comparison direction, XLGB showed greater improvement in lumbar spine BMD than TMK (MD 0.05, 95% CI 0.01–0.09), and GSK (MD 0.07, 95% CI -0.02–0.12) (Supplementary Appendix Table S7.1). CINeMA results rated the overall evidence quality for lumbar spine BMD as very low to moderate (Supplementary Appendix Table S8.1).

**FIGURE 2:**
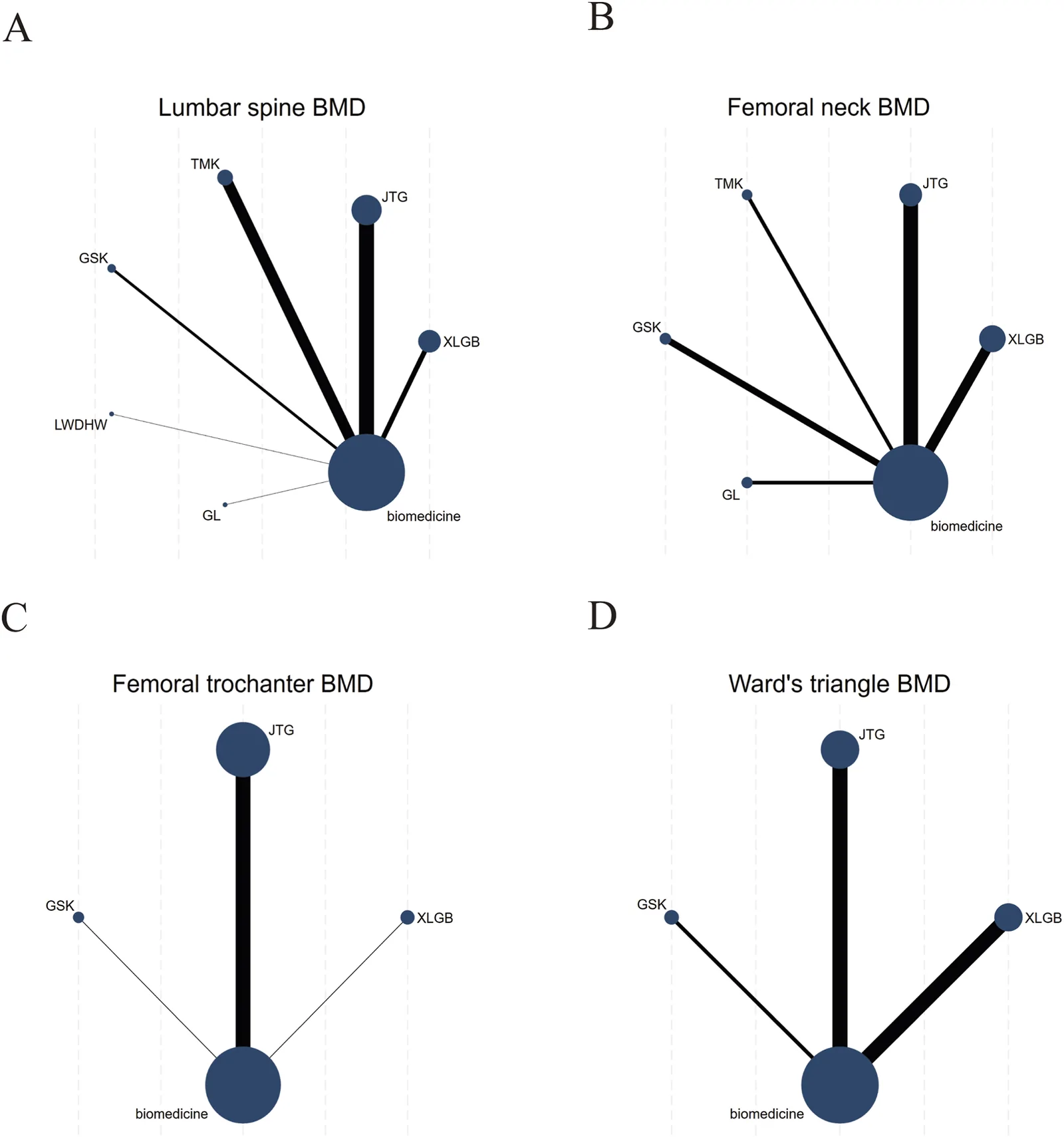
Network plot for direct comparisons of BMD. Note: This network illustrates the available comparative data for CCPPs + biomedicine versus biomedicine in diabetic osteoporosis. Node sizes are proportional to the number of trial participants, and the thickness of the lines connecting nodes is proportional to the number of randomly assigned participants in direct comparisons of the two treatment regimens. The figure features four subfigures, each representing a different outcome: **(A)** Lumbar spine bone mineral density; **(B)** Femoral neck bone mineral density; **(C)** Femoral trochanter bone mineral density; **(D)** Ward’s triangle bone mineral density.

**FIGURE 3:**
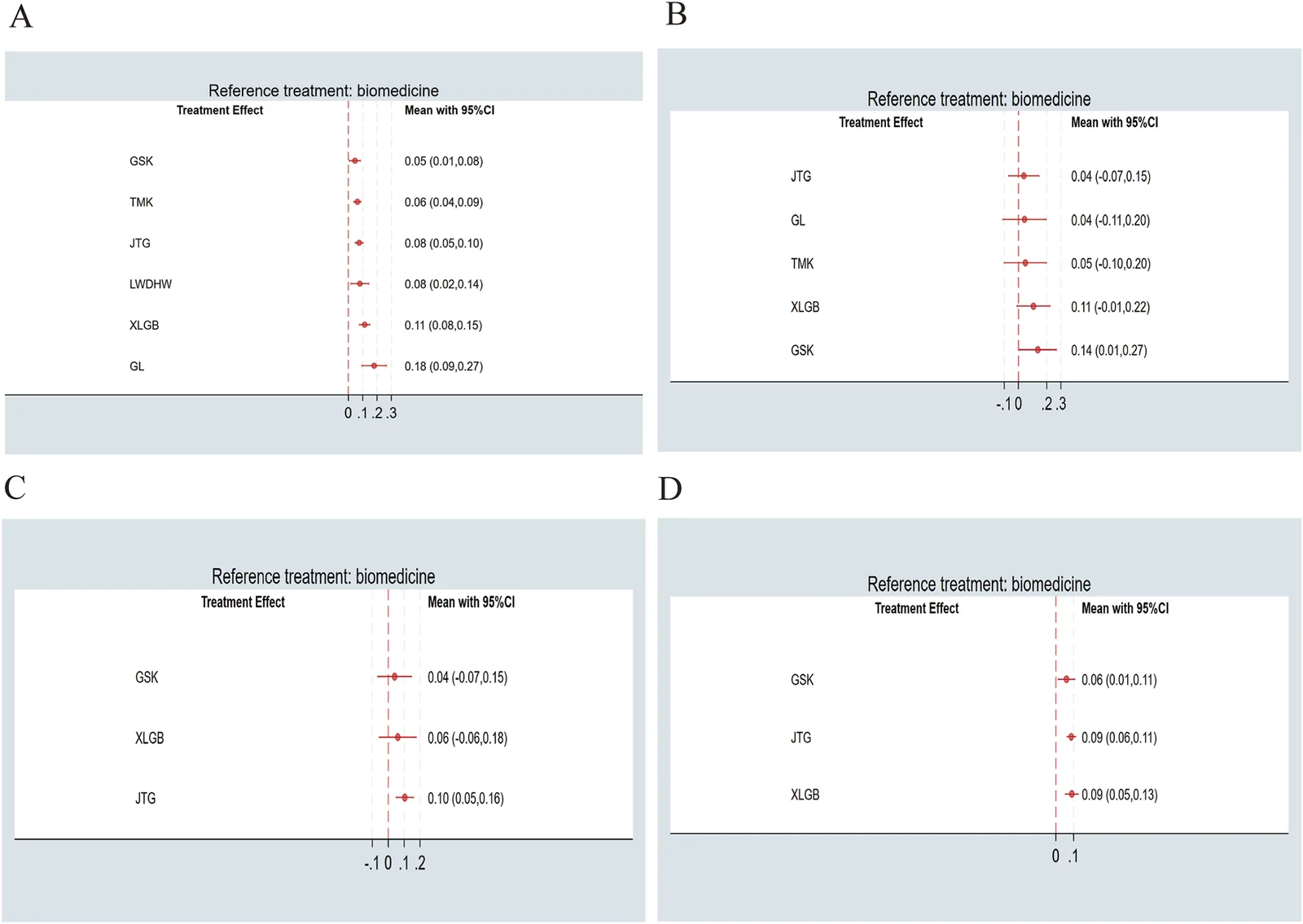
Forest plot for direct comparisons of BMD.Note: Forest plot of the network effect size between CCPPs + biomedicine and biomedicine in BMD.The figure features four subfigures, each representing a different outcome: **(A)** Lumbar spine bone mineral density; **(B)** Femoral neck bone mineral density; **(C)** Femoral trochanter bone mineral density; **(D)** Ward’s triangle bone mineral density.

#### 3.4.2 Femoral neck bone mineral density

The NMA for femoral neck BMD included 21 trials involving 2,296 participants. Five CCPP regimens combined with biomedicine were available for indirect comparisons (Figure 2B). As shown in the forest plot (Figure 3B), compared with biomedicine alone, all five CCPP regimens increased femoral neck BMD, but only GSK + biomedicine reached statistical significance [MD = 0.14, 95% CI (0.01, 0.27), SUCRA = 81.8%; low confidence of evidence]. The next ranked interventions were XLGB + biomedicine [MD = 0.11, 95% CI (−0.01, 0.22), SUCRA = 72.2%; low confidence of evidence] and TMK + biomedicine [MD = 0.05, 95% CI (−0.10, 0.20), SUCRA = 45.8%; very low confidence of evidence]. No statistically significant differences were found in pairwise comparisons among the five CCPPs (Supplementary Appendix Table S7.2). CINeMA results rated the overall evidence quality for femoral neck BMD as very low to low (Supplementary Appendix Table S8.2).

#### 3.4.3 Femoral trochanter bone mineral density

The NMA for femoral trochanter BMD included 7 trials involving 832 participants. Three CCPP regimens were available for indirect comparisons (Figure 2C). As shown in the forest plot (Figure 3C), compared with biomedicine alone, all three CCPP regimens significantly increased femoral trochanter BMD. JTG + biomedicine showed the most significant effect [MD = 0.10, 95% CI (0.05, 0.16), SUCRA = 86.5%; low confidence of evidence]. No statistically significant differences were found in pairwise comparisons (Supplementary Appendix Table S7.3). CINeMA results rated the overall evidence quality for femoral trochanter BMD as very low to low (Supplementary Appendix Table S8.3).

#### 3.4.4 Ward’s triangle bone mineral density

The NMA for Ward’s triangle BMD included 5 trials involving 621 participants. Three CCPP regimens were available for indirect comparisons (Figure 2D). As shown in the forest plot (Figure 3D), compared with biomedicine alone, all three CCPP regimens significantly increased Ward’s triangle BMD. XLGB + biomedicine showed the largest effect [MD = 0.09, 95% CI (0.05, 0.13), SUCRA = 80.0%; low confidence of evidence], followed by JTG + biomedicine [0.09 (0.06, 0.11), 76.1%] and GSK [0.06 (0.01, 0.11), 43.7%]. No statistically significant differences were found in pairwise comparisons (Supplementary Appendix Table S7.4). CINeMA results rated the overall evidence quality for Ward’s triangle BMD as very low to low (Supplementary Appendix Table S8.4).

#### 3.4.5 Osteocalcin

Analysis of osteocalcin levels focused on six regimens combined with biomedicine (Figure 4A). The forest plot (Figure 5A) showed that compared with biomedicine alone, five of the six combination regimens significantly increased osteocalcin levels. Notably, JTG and GL combined with biomedicine showed statistically significant superiority over biomedicine alone. In terms of efficacy ranking, JTG + biomedicine [1.80, (1.21, 2.39), 96.6%, moderate confidence of evidence] was most effective for increasing osteocalcin, followed by GL + biomedicine [MD 0.99, 95% CI 0.16, 1.83, low confidence of evidence]. JTG + biomedicine was statistically superior to TMK + biomedicine (MD 1.18, 95% CI 0.00, 2.35), LWDHW + biomedicine (MD 1.45, 95% CI 0.26, 2.65), and XLGB + biomedicine (MD -2.63, 95% CI -4.15, −1.10). XLGB + biomedicine was the least effective (Supplementary Appendix Table S7.5). According to CINeMA, the overall evidence certainty was rated as very low to moderate (Supplementary Appendix Table S8.5).

**FIGURE 4:**
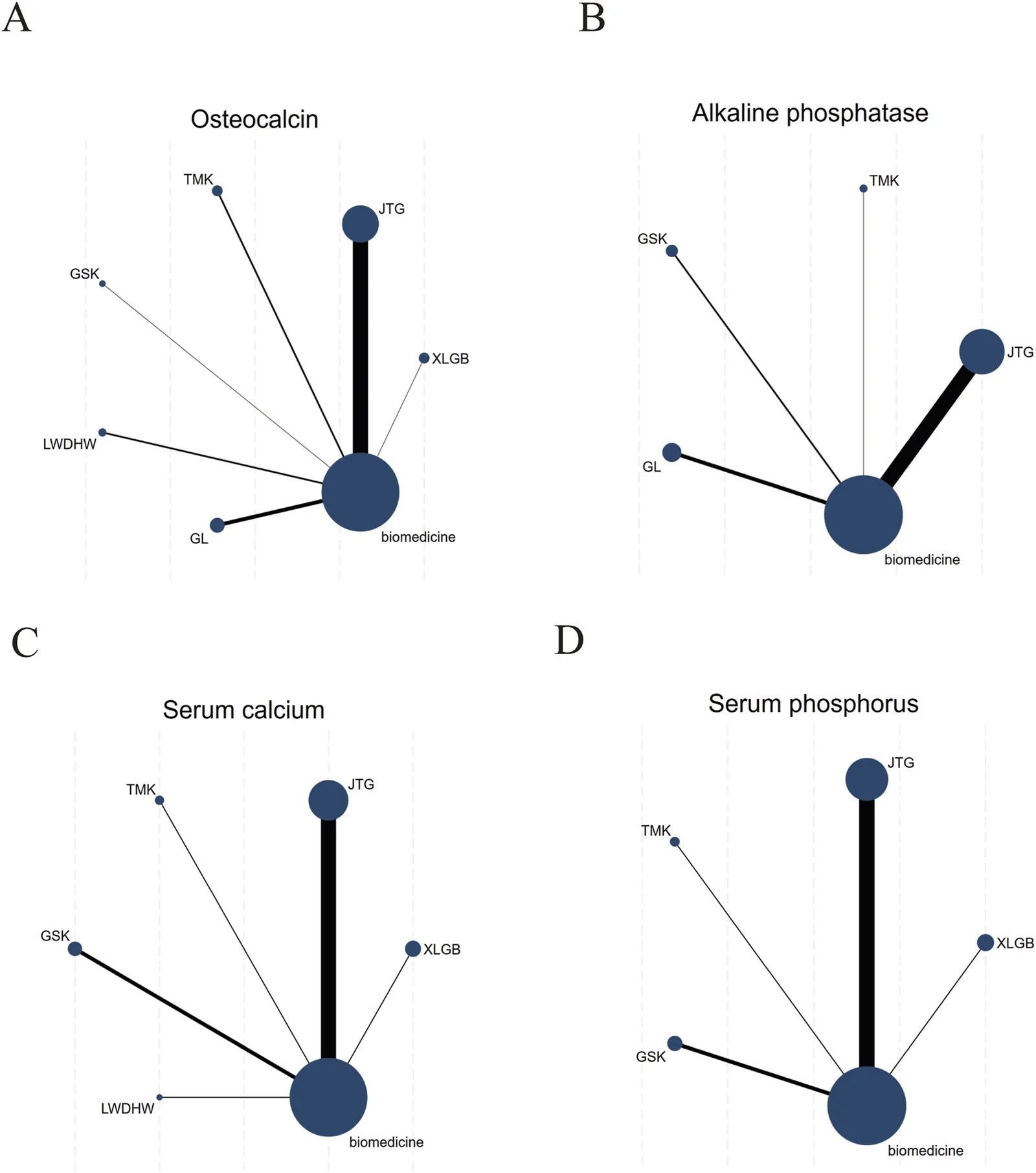
Network plot for direct comparisons of bone turnover markers. Note: This network illustrates the available comparative data for CCPPs + biomedicine versus biomedicine in diabetic osteoporosis. Node sizes are proportional to the number of trial participants, and the thickness of the lines connecting nodes is proportional to the number of randomly assigned participants in direct comparisons of the two treatment regimens. The figure features four subfigures, each representing a different outcome: **(A)** Osteocalcin; **(B)** Alkaline phosphatase; **(C)** Serum calcium; **(D)** Serum phosphorus.

**FIGURE 5:**
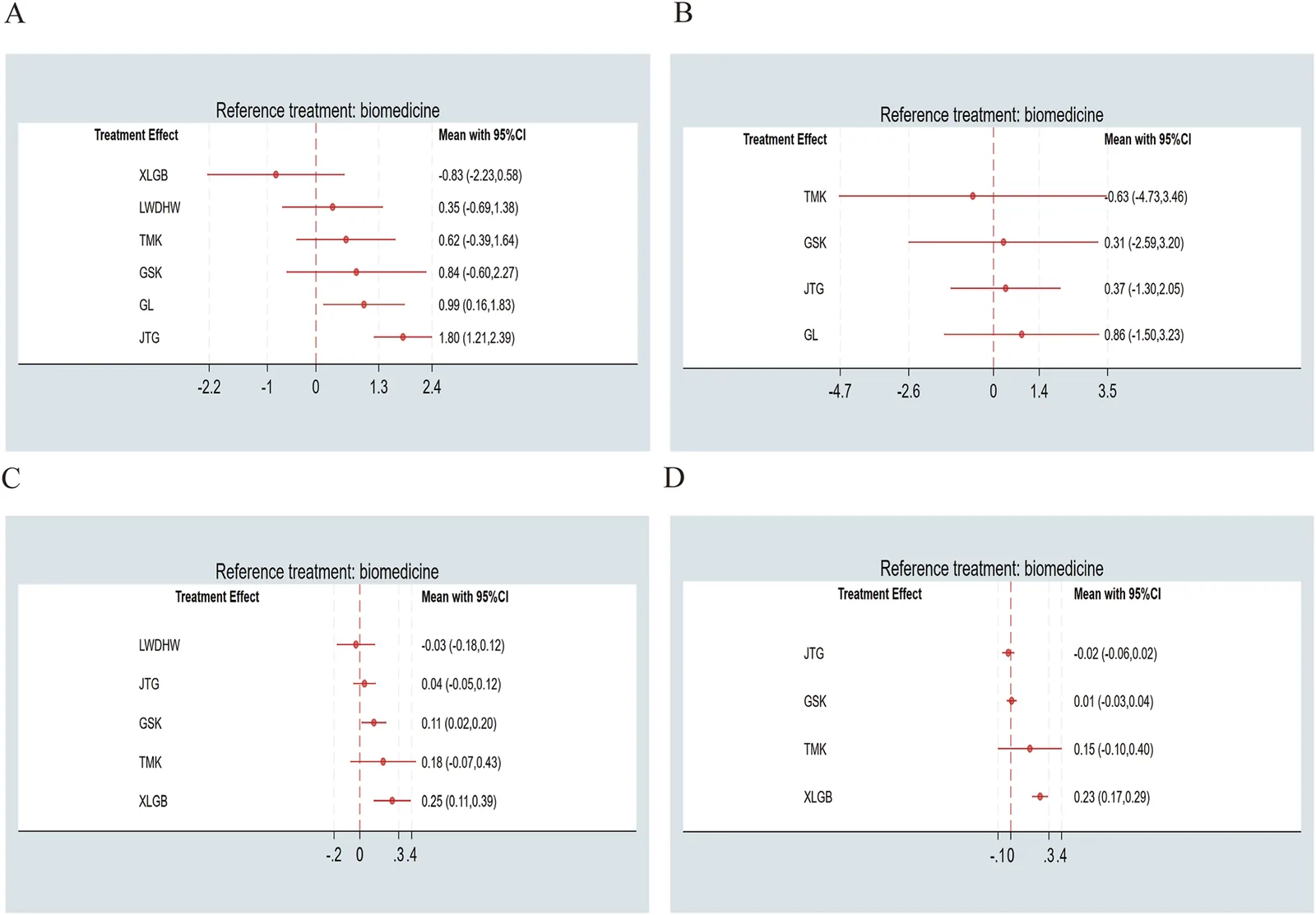
Forest plot for direct comparisons of bone turnover markers. Note: Forest plot of the network effect size between CCPPs + biomedicine and biomedicine in bone turnover markers.The figure features four subfigures, each representing a different outcome: **(A)** Osteocalcin; **(B)** Alkaline phosphatase; **(C)** Serum calcium; **(D)** Serum phosphorus.

#### 3.4.6 Alkaline phosphatase

Analysis of alkaline phosphatase levels focused on four regimens combined with biomedicine, involving 1,309 participants (Figure 4B). The forest plot (Figure 5B) showed that compared with biomedicine alone, only TMK + biomedicine reduced alkaline phosphatase levels [MD -0.63, (−4.73, 3.46), 67.2%, very low confidence of evidence], but the difference was not statistically significant. No significant differences were found in pairwise comparisons (Supplementary Appendix Table S7.6). CINeMA results rated the overall evidence quality for alkaline phosphatase as very low to moderate (Supplementary Appendix Table S8.6).

#### 3.4.7 Calcium and phosphorus

For serum calcium levels, the NMA included nine studies comparing five regimens combined with biomedicine, involving 1,079 participants (Figure 4C). The forest plot (Figure 5C) showed that compared with biomedicine alone, four of the five combination regimens increased serum calcium levels. XLGB, TMK, GSK, and JTG combined with biomedicine were superior to biomedicine alone, but only XLGB [0.25, (0.11, 0.39), 92.1%, low confidence of evidence] and GSK [0.11, (0.02, 0.20), 63.2%, low confidence of evidence] reached statistical significance. In pairwise comparisons, XLGB + biomedicine was superior to JTG + biomedicine (MD 0.21, 95% CI 0.05, 0.38) and LWDHW + biomedicine (MD 0.28, 95% CI 0.08, 0.48) (Supplementary Appendix Table S7.7). CINeMA rated the overall evidence quality for serum calcium as very low to low (Supplementary Appendix Table S8.7).

For serum phosphorus levels, the NMA included eight studies comparing four regimens combined with biomedicine, involving 1,019 participants (Figure 4D). The forest plot (Figure 5D) showed that compared with biomedicine alone, GSK, TMK, and XLGB combination regimens increased serum phosphorus levels. XLGB [0.23, (0.17, 0.29), 93.4%, high confidence of evidence] showed a statistically significant increase. In pairwise comparisons, XLGB + biomedicine was superior to JTG + biomedicine (MD 0.25, 95% CI 0.18, 0.33) and GSK + biomedicine (MD 0.22, 95% CI 0.15, 0.29) (Supplementary Appendix Table S7.8). CINeMA rated the overall evidence quality for serum phosphorus as very low to high (Supplementary Appendix Table S8.8).

#### 3.4.8 Fasting blood glucose

The NMA for fasting blood glucose included nine trials comparing four regimens combined with biomedicine, involving 798 participants (Figure 6A). The forest plot (Figure 6B) showed that compared with biomedicine alone, LWDHW, JTG, and GSK combination regimens significantly reduced fasting blood glucose levels. LWDHW [−1.03, (−1.70, −0.35), 86.1%, low confidence of evidence] was most effective, followed by JTG [−0.65, (−1.48, 0.17), 65.8%] and GSK [−0.38, (−1.88, 1.12), 48.5%]. No significant differences were found in pairwise comparisons (Supplementary Appendix Table S7.9). CINeMA rated the overall evidence quality for fasting blood glucose as very low to low (Supplementary Appendix Table S8.9).

**FIGURE 6:**
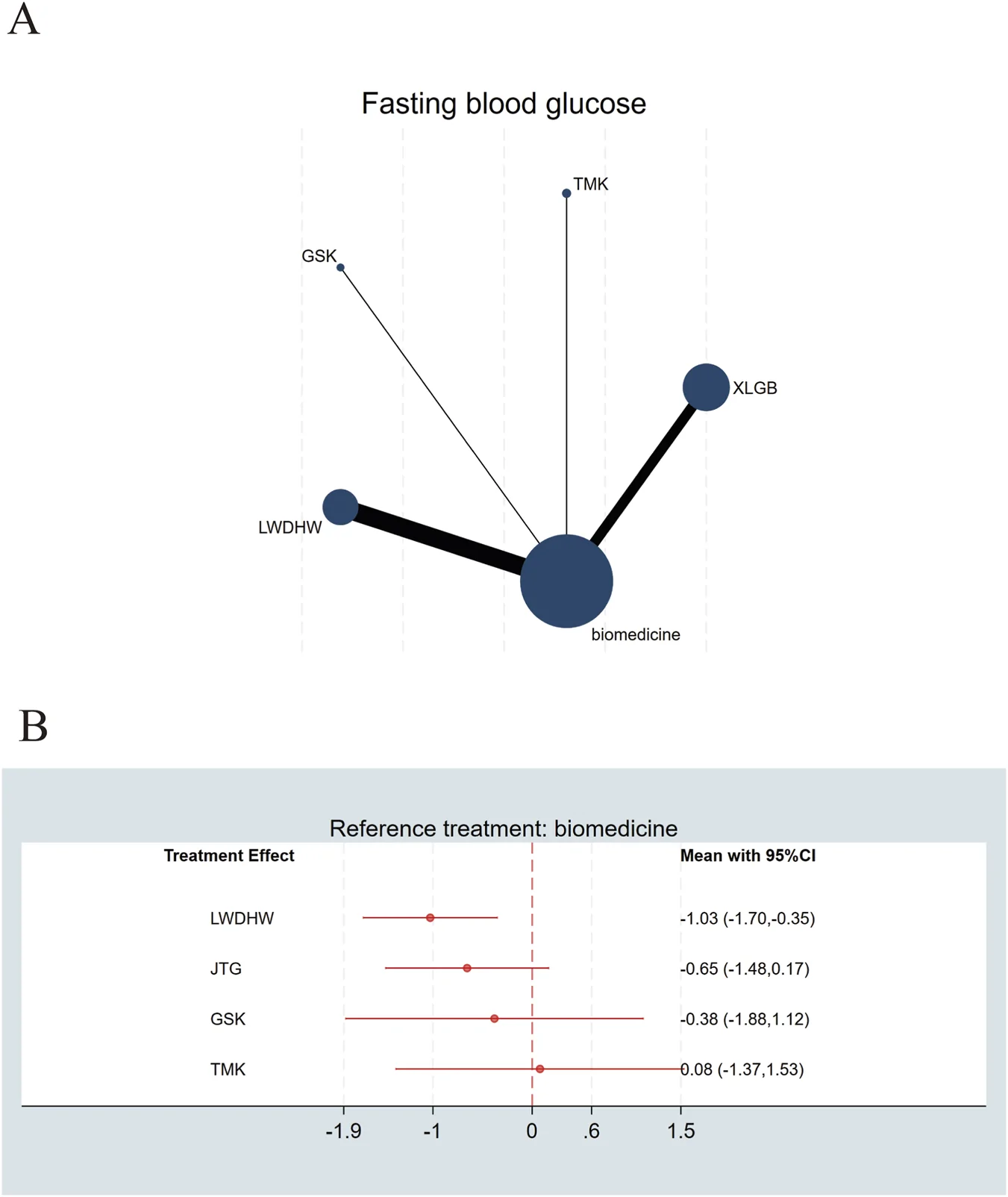
**(A)** Network plot for direct comparisons of Fasting blood glucose; **(B)** Forest plot for direct comparisons of Fasting blood glucose.

#### 3.4.9 Total effective rate

The NMA for total effective rate (TER) included 22 trials comparing six regimens combined with biomedicine, involving 2,519 participants (Figure 7A). The criteria used to define treatment response were not uniform across studies and generally incorporated changes in BMD, pain or VAS scores, TCM syndrome scores, or improvement in clinical symptoms. As shown in the forest plot (Figure 7B), compared with biomedicine alone, all six treatment regimens significantly increased the TER. According to SUCRA rankings, the top three interventions were: GSK [RR 1.68 (1.22, 2.32), 96.2%, high confidence of evidence], LWDHW [1.35 (1.14, 1.61), 78.6%, high confidence of evidence], and TMK [1.24 (1.14, 1.34), 62.2%, moderate confidence of evidence]. League table analysis showed that XLGB + biomedicine (RR 0.68, 95% CI 0.49, 0.94) and JTG + biomedicine (RR 0.68, 95% CI 0.49, 0.94) were statistically inferior to GSK + biomedicine (Supplementary Appendix Table S7.10). CINeMA rated the overall evidence quality for TER as low to high (Supplementary Appendix Table S8.10).

**FIGURE 7:**
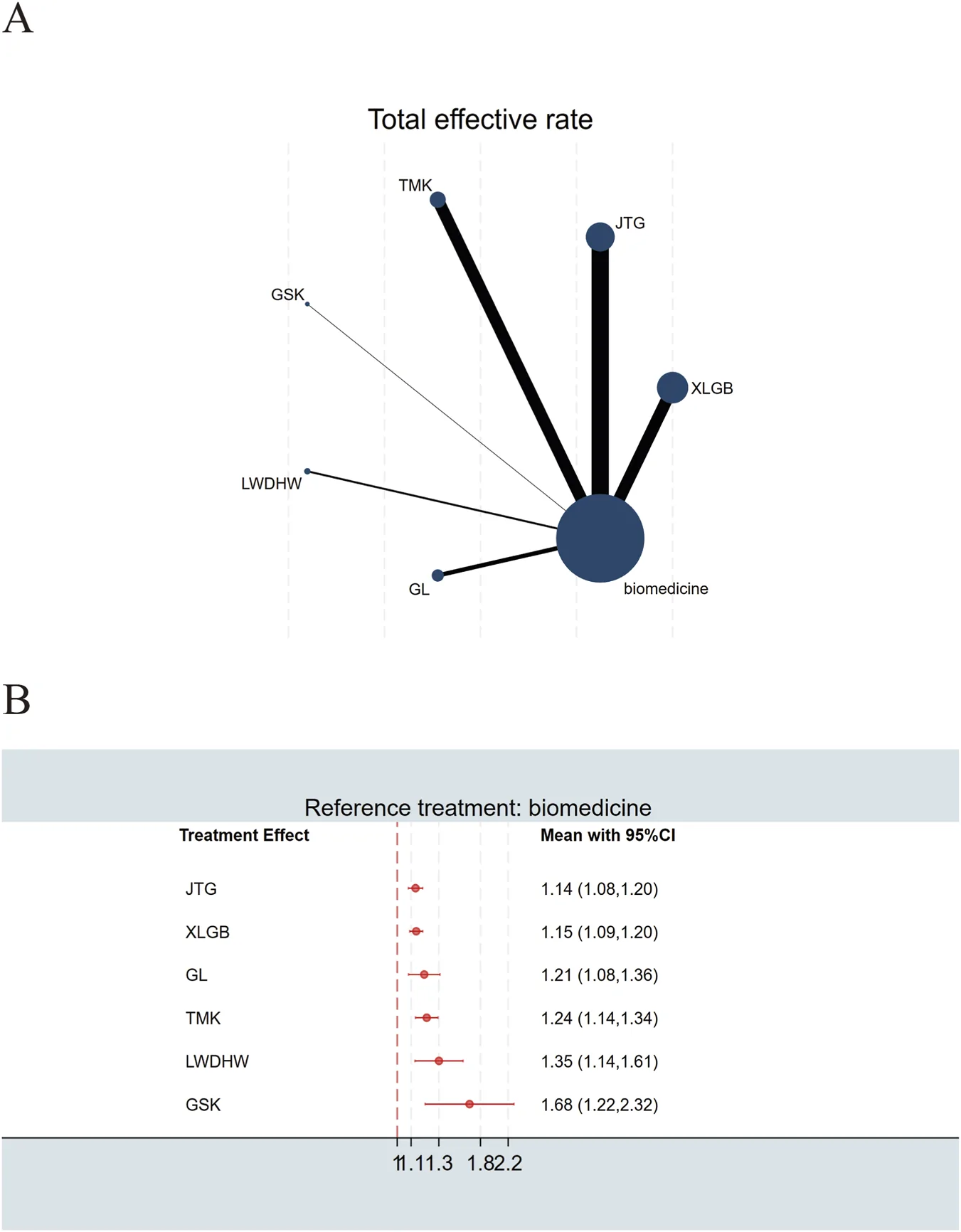
**(A)** Network plot for direct comparisons of Total effective rate; **(B)** Forest plot for direct comparisons of Total effective rate.

#### 3.4.10 Adverse events

The NMA also assessed adverse events (AEs) when combining CCPPs with biomedicine compared with biomedicine alone (Figure 8A). As shown in the forest plot (Figure 8B), five CCPPs combined with biomedicine showed a lower risk of AEs, but none reached statistical significance. According to SUCRA rankings, the top three interventions were: GL + biomedicine (SUCRA 81.1%, low confidence of evidence), TMK + biomedicine (SUCRA 71.0%, very low confidence of evidence), and XLGB + biomedicine (SUCRA 58.2%, low confidence of evidence). No significant differences were found in pairwise comparisons (Supplementary Appendix Table S7.11). CINeMA rated the overall evidence quality for adverse events as very low to low (Supplementary Appendix Table S8.11).

**FIGURE 8:**
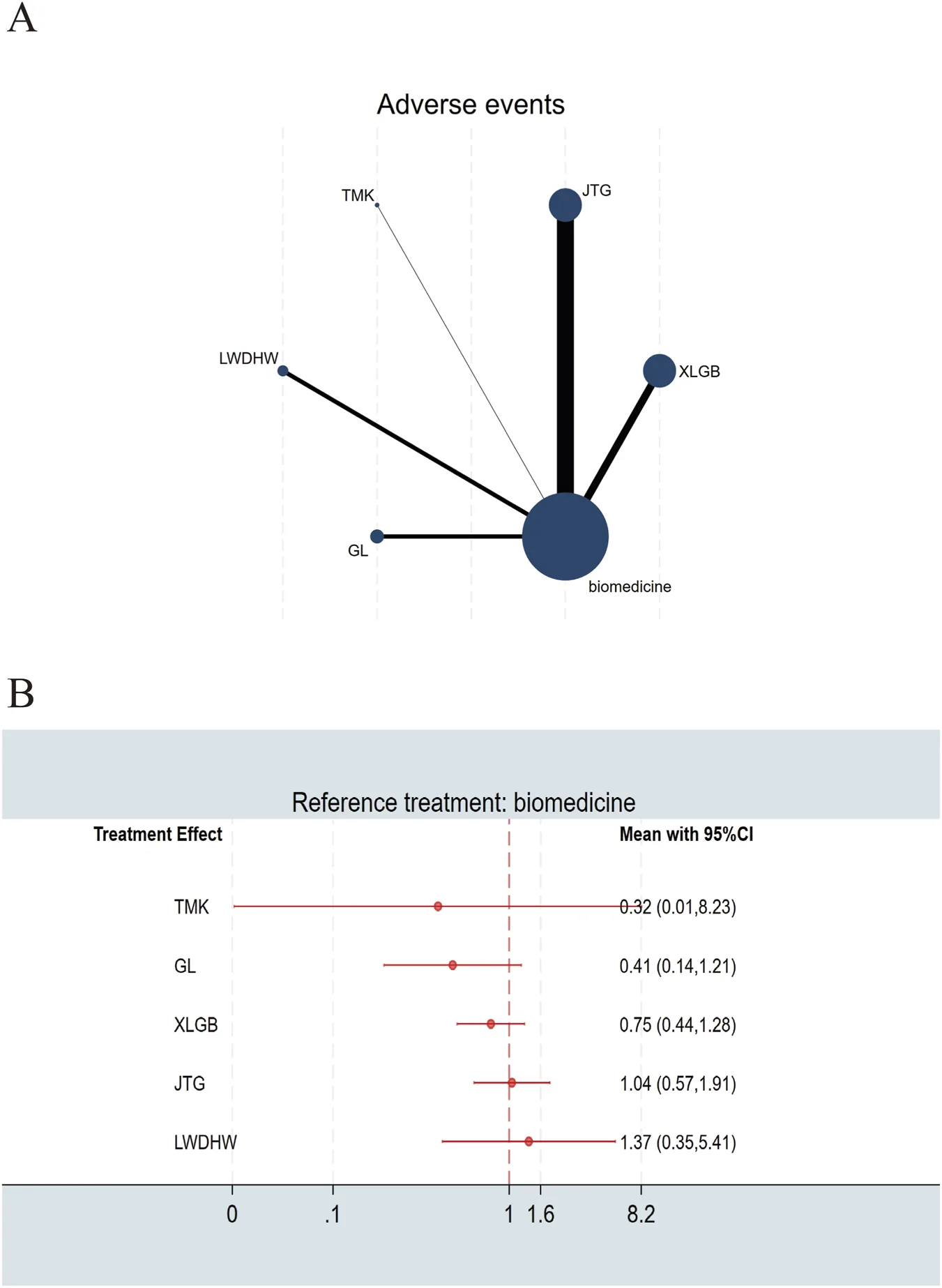
**(A)** Network plot for direct comparisons of Adverse events; **(B)** Forest plot for direct comparisons of Adverse events.

## 4 Discussion

### 4.1 Summary of main findings

This network meta-analysis systematically evaluated the efficacy of six commercial Chinese polyherbal preparations (CCPPs) for diabetic osteoporosis. A total of 34 RCTs involving 3,720 patients were included. The main outcomes assessed included lumbar spine BMD, femoral neck BMD, femoral trochanter BMD, Ward’s triangle BMD, osteocalcin, alkaline phosphatase, calcium, phosphorus, fasting blood glucose, TER, and adverse events. Compared with biomedicine alone, CCPPs combined with biomedicine showed benefits across several outcomes, including TER, BMD, bone turnover markers, and fasting blood glucose. Specifically, SUCRA rankings suggested that GSK had the highest relative ranking for TER; XLGB, JTG and GSK showed potential advantages for BMD; JTG ranked highest for osteocalcin; XLGB ranked highest for calcium and phosphorus; LWDHW ranked highest for fasting blood glucose, and GL had the lowest risk of adverse events. These rankings should be interpreted cautiously, particularly when supported by low or very low certainty evidence, and should be considered exploratory rather than definitive evidence of comparative superiority. Overall, CCPPs combined with biomedicine showed potential clinical benefits compared with biomedicine alone. The available patient characteristics showed broad clinical overlap across treatment comparisons, although some variation was observed in age, sex distribution, and diabetes duration. However, variations in treatment duration and background biomedicine regimens may have contributed to clinical heterogeneity and should be considered when interpreting the comparative effects. Baseline BMD was not consistently reported, which limited detailed comparison of baseline disease severity. Therefore, transitivity was considered broadly plausible based on the available study level data, although residual uncertainty cannot be excluded.

### 4.2 Pharmacological mechanisms of TCM in the context of modern medicine

The key components of the CCPPs in this NMA demonstrate significant therapeutic potential by regulating multiple biological processes, including promoting osteogenic differentiation, inhibiting osteoclastogenesis, suppressing inflammatory responses, and counteracting oxidative stress. The mechanisms of action of these components often involve modulation of various cell signaling pathways.

Icariin, the main component of Epimedium, exhibits excellent dual-directional regulatory functions, i.e., promoting osteogenesis while inhibiting osteoclastogenesis. Icariin activates the Wnt/β-catenin pathway, increases cytoplasmic β-catenin levels and promotes its nuclear translocation, inducing the expression of osteogenic factors such as Runx2 and Osterix. At the same time, it enhances the osteogenic differentiation potential of bone marrow mesenchymal stem cells (BMSCs) by activating the Hippo-Yap/TAZ pathway (Fu et al., 2025). In terms of osteoclast regulation, icariin blocks osteoclast formation by modulating the ratio of osteoprotegerin (OPG) to receptor activator of nuclear factor-κB ligand (RANKL). For diabetes-specific damage, icariin activates the Nrf2/ARE pathway to induce antioxidant enzyme production and neutralize reactive oxygen species (ROS) (Hu et al., 2025b).

The active components of Salvia miltiorrhiza include lipophilic tanshinone IIA and water-soluble salvianolic acid B. Salvia miltiorrhiza activates the HIF-1α/VEGF and Slit3 signaling axes, significantly enhancing angiogenesis within bone tissue and providing necessary nutritional support for osteoblasts (Ye et al., 2023). Salvianolic acid B has been shown to promote osteogenic differentiation of hMSCs by activating the ERK pathway. Tanshinone IIA is a natural inhibitor of cathepsin K, blocking the degradation of bone collagen by osteoclasts (Wang L. et al., 2024). At the same time, it reduces inflammatory bone destruction by downregulating the MAPK/NF-κB pathway (Hu et al., 2025a).

Catalpol, a core component of Prepared Rehmannia Root, has multiple mechanisms in treating T2DOP. Catalpol inhibits the histone demethylase KDM7A. Under high-glucose conditions, inhibition of KDM7A relieves the epigenetic suppression of the Wnt/β-catenin pathway, thereby restoring osteogenic differentiation capacity (Cheng et al., 2020). Catalpol induces apoptosis of mature osteoclasts through the SIRT6-ERα-FasL signaling cascade, reducing high bone turnover in the diabetic state (Li N. et al., 2025). Rehmannia glutinosa polysaccharide (RGP) improves systemic inflammation and alleviates diabetic bone loss by upregulating HDAC6 mRNA expression in regulatory T cells (Tregs) (Yue et al., 2024).

Extracts of Psoralea corylifolia, such as psoralen and corylin, have defined molecular targets. Corylin directly binds to recombination activating gene 1 (RAG1). Activation of RAG1 suppresses local and systemic inflammation in the diabetic environment and directly promotes BMSCs differentiation toward osteoblasts (Lin et al., 2026). Psoralen acts on the bone morphogenetic protein (BMP) signaling pathway, increasing BMP-2 expression and activating downstream Smad cascades, thereby enhancing alkaline phosphatase (ALP) activity (Huang et al., 2021).

Astragalus membranaceus extract significantly inhibits AGEs-induced macrophage polarization, reducing secretion of TNF-α and IL-1β by blocking the p38 MAPK and NF-κB pathways (Qin et al., 2012). Astragalus upregulates Klotho protein and vitamin D receptor (VDR) expression, improving calcium-phosphorus metabolism. Astragaloside IV induces BMSC differentiation toward osteoblasts through the NMUR2-mediated Wnt/β-catenin pathway (Chai et al., 2021).

Codonopsis pilosula extract inhibits NLRP3 inflammasome and caspase-1 activation, alleviating high glucose-induced osteoblast pyroptosis and restoring Wnt/β-catenin pathway activity. The monomeric lobetyolin from Codonopsis inhibits NF-κB pathway by blocking p50/p65 nuclear translocation, thereby reducing bone loss (Xiu et al., 2025).

### 4.3 Pharmacological mechanisms of CCPPs in the modern medical context

In this NMA, GSK, GL, XLGB, JTG, LWDHW, and TMK combined with biomedicine showed potential benefits in BMD, calcium and phosphorus levels, and blood glucose control. Differences in their relative rankings may partly reflect their distinct multitarget pharmacological mechanisms, although these rankings should not be interpreted as definitive evidence of superiority.

The GSK regimen ranked highest in TER. Gushukang capsule, recorded in the Chinese Pharmacopoeia, is a traditional clinical prescription for osteoporosis (Li et al., 2001). Its main ingredients include Epimedium, Prepared Rehmannia Root, Drynaria, Astragalus, Salvia miltiorrhiza, black fungus, and cucumber seed. Previous studies have shown that GSK can increase BMD and restore trabecular bone microstructure in ovariectomized (OVX) mice (Wang et al., 2018). Icariin may alleviate estrogen deficiency-induced osteoporosis by promoting IGF-I signaling in bone (Zhou et al., 2021). Additionally, GSK may improve osteoporosis by regulating vitamin D metabolism and calcium homeostasis (Li et al., 2020).

GL is mainly composed of Epimedium, Cibotii Rhizoma, Achyranthes bidentata, Clematis chinensis, Chaenomeles speciosa, Curcuma longa, Psoralea corylifolia, Codonopsis pilosula, and Pueraria lobata. It has the effects of strengthening bones, dispelling wind-dampness, activating blood and removing stasis, and relieving pain, and is used for lumbago and leg pain caused by wind-cold-dampness and osteoporosis. As demonstrated by both *in vivo* and *in vitro* experiments, icariin activates the Wnt/β-catenin signaling pathway, upregulates the expression of Runx2 and ALP, drives the osteogenic differentiation of mesenchymal stem cells, effectively lowers blood glucose levels, and inhibits osteoclastic activity (Qi et al., 2019). Meanwhile, psoralen blocks the fusion and proliferation of osteoclasts by modulating the OPG/RANKL ratio and activating oestrogen receptor (ER-α) (Chen et al., 2017). This multicomponent, multitarget network synergy not only compensates for the potential risk of calcium-phosphorus metabolic derangement associated with conventional glucose-lowering agents but also exhibits excellent clinical efficacy and safety in improving bone mineral density and reducing gastrointestinal adverse reactions (Tan et al., 2024).

XLGB is a compound formula containing Epimedium, Dipsacus, Salvia miltiorrhiza, Anemarrhena asphodeloides, Psoralea corylifolia, and Rehmannia glutinosa. According to TCM theory, it nourishes the liver and kidney, promotes blood circulation, and strengthens bones (Yan et al., 2026). A meta-analysis found that XLGB showed better overall clinical efficacy, significantly improved BMD at the lumbar spine and femoral neck, and increased serum osteocalcin levels, with no serious adverse events reported (Yang et al., 2024). Modern pharmacological studies have confirmed the preventive and therapeutic effects of XLGB on diabetes-related osteoporosis. XLGB downregulates RANKL mRNA expression and upregulates OPG mRNA, thereby inhibiting osteoclast activity and promoting the differentiation and mineralization of MC3T3-E1 osteoblast cells (Wu et al., 2017).

JTG is a synthetic tiger bone powder made from various farmed animal bones, used as a substitute for natural tiger bone in osteoporosis. JTG prevented bone loss and improved bone microstructure in OVX rat models. At the same time, JTG promotes osteogenic differentiation of BMSCs *in vitro* by regulating the BMP and Wnt/β-catenin pathways and inhibits osteoclastogenesis by suppressing the NF-κB pathway (Shen et al., 2022). A systematic review showed that JTG has significant effects in relieving pain, improving BMD, and preventing fractures in patients with primary osteoporosis (Li W. et al., 2025).

LWDHW is a traditional Chinese formula composed of six herbs: Prepared Rehmannia Root, Cornus officinalis (processed), Dioscorea opposita, Paeonia suffruticosa, Poria, and Alisma orientale. It is used to treat various conditions related to kidney yin deficiency, such as dizziness, tinnitus, soreness and weakness of the waist and limbs, and sweating (Zhou et al., 2016). A clinical trial showed that LWDHW effectively reduced bone turnover markers, including serum procollagen type I N-terminal propeptide (P1NP) and β-isomerized C-terminal telopeptide (β-CTX), increased BMD at the lumbar spine and hip, and significantly improved patients’ quality of life (Zhao et al., 2024).

TMK is composed of 11 TCM herbs, mainly including Astragalus, Rehmannia glutinosa, Paeonia rubra, Salvia miltiorrhiza, Achyranthes bidentata, Ophiopogon japonicus, Polygonatum sibiricum, Epimedium, and Pueraria lobata. It has the effects of nourishing yin and clearing heat, activating blood and removing stasis, supplementing qi and strengthening the kidney, and has shown good efficacy in treating diabetes patients with osteoporosis. Studies have shown that TMK promotes osteoblast synthesis, improves BMD, and alleviates lower back pain symptoms of osteoporosis. Animal studies have shown that total saponins of Achyranthes bidentata reduce urinary hydroxyproline excretion, upregulate alkaline phosphatase and osteocalcin expression levels in OVX rat models, and promote bone mass gain (He et al., 2014). Related studies also indicate that Epimedium significantly promotes osteoblast differentiation (Indran et al., 2016). Additionally, research has pointed out that the isoflavones in Pueraria lobata inhibit cellular oxidative stress, thereby promoting osteoblast differentiation and inhibiting osteoclastogenesis, which is important for improving BMD (Tang et al., 2012).

### 4.4 Treatment of diabetic osteoporosis with CCPPs: clinical value and future directions

The pathogenesis of DOP is far more complex than that of primary osteoporosis, involving hyperglycemic toxicity, insulin resistance, oxidative stress, and abnormal accumulation of AGEs in the bone matrix. This pathological remodeling creates a “high BMD–high fracture risk” paradox unique to diabetic bone disease, where bone microstructure and mechanical properties have already significantly deteriorated despite normal or even increased BMD. Existing anti-osteoporosis drugs, such as bisphosphonates and denosumab, mainly inhibit bone resorption. Although they can increase bone mass, their improvement on the core issue of bone fragility is limited. Moreover, some glucose-lowering drugs (e.g., thiazolidinediones) increase fracture risk, making it difficult to achieve both glycemic control and bone protection goals. This “single-target, single-dimensional” intervention model creates an objective need for the “multi-target, holistic regulation” approach of CCPPs. The included studies showed improvements in both BMD and blood glucose with CCPPs combined with biomedicine, suggesting a potential role in the simultaneous regulation of glucose and bone metabolism. DOP has a long course, and medication safety is key to adherence. Long-term use of bisphosphonates carries risks such as gastrointestinal reactions and osteonecrosis of the jaw, whereas the adverse reactions in the CCPPs groups included in this study were mostly mild gastrointestinal discomfort that resolved with symptomatic treatment or dose reduction, indicating overall high safety. This good long-term tolerability has important clinical value for patients with chronic diseases requiring lifelong management.

Although these findings are encouraging, several challenges still hinder the widespread application of CCPPs. Importantly, the current clinical evidence is mainly derived from Chinese populations and healthcare settings, where CCPPs are routinely used as complementary therapies alongside biomedicine. Therefore, the clinical applicability of these findings to other ethnic populations and healthcare systems remains uncertain and requires further validation. To date, most mechanistic studies are limited to laboratory or animal models, and there remains a lack of large-scale, high-quality clinical trials to verify their effectiveness and safety in diverse human populations. The complex multi-component nature of CCPPs also makes it difficult to identify specific active ingredients or fully understand their mechanisms of action. Moreover, the lack of standardization in TCM syndrome differentiation is an important reason affecting the generalizability of results. Different studies have varying inclusion criteria for “kidney deficiency with blood stasis” or “spleen-kidney deficiency,” and the selection of CCPPs corresponding to syndrome types is often arbitrary, which may lead to confounding from “formula-syndrome mismatch” and reduce the precision of effect estimates.

To promote the clinical application of CCPPs in diabetic osteoporosis, it is recommended to design multicenter, large-sample, double-blind, double-dummy RCTs and extend follow-up periods. Using multi-omics technologies such as metabolomics, proteomics, and gut microecology, the molecular network through which CCPPs formulas influence bone metabolism by improving insulin signaling, regulating bone immunity, and modulating gut metabolites should be elucidated, advancing from empirical use to precision targeted therapy. Additionally, CCPPs showing potential benefits in this network meta-analysis may provide preliminary evidence for future clinical decision-making and guideline development. However, their incorporation into clinical practice should consider the strength of available evidence, local healthcare contexts, treatment accessibility, and patient characteristics. Further evidence from rigorously designed studies is required before establishing broader recommendations.

### 4.5 Limitations

This study applied network meta-analysis for the first time to evaluate the effectiveness of six CCPPs combined with biomedicine for managing diabetic osteoporosis. The results provide important references for selecting integrated traditional Chinese and biomedicine treatment strategies. This study has the following limitations:

First, clinical heterogeneity existed in the comparator interventions and some potential effect modifiers. Biomedicine was not standardized across the included RCTs and included different background therapies, such as bisphosphonates, active vitamin D analogues, calcium and vitamin D supplementation, and glucose lowering therapy. The specific agents, doses, frequencies, and treatment durations also varied among studies. Because biomedicine represented background clinical management rather than an independent intervention of interest, these variations may contribute to between study heterogeneity and potentially affect the magnitude and precision of pooled effect estimates. The transitivity assessment considered age, sex distribution, diabetes duration, baseline disease severity as reflected by baseline BMD where available, treatment duration, and background biomedicine. Although the available patient characteristics showed some overlap across treatment comparisons, several variables were incompletely reported. In particular, baseline BMD was not consistently available, limiting the assessment of baseline disease severity, while differences in treatment duration and background biomedicine may have introduced additional variability in treatment effects across studies. Subgroup analyses according to specific Western medications were not performed because the available studies did not provide sufficient and balanced comparisons across individual biomedicine categories. Therefore, although transitivity was considered broadly plausible, residual uncertainty remains. Future studies should standardize background treatment regimens where possible and report baseline characteristics, disease severity, and treatment details more consistently.

Second, the overall methodological quality of the included studies was limited, and the certainty of evidence for many network comparisons was low or very low. This uncertainty may reduce the robustness of comparative estimates and affect the stability of treatment rankings. A sensitivity analysis excluding studies with high risk of bias was not performed because several network comparisons were supported by a limited number of trials, and excluding these studies could reduce network connectivity and produce unstable estimates. This limitation should be considered when interpreting the robustness of the findings. SUCRA values reflect relative ranking probabilities and should not be interpreted independently of effect estimates, confidence intervals, and certainty of evidence. Therefore, the ranking results in this NMA should be regarded as exploratory and interpreted with caution.

Third, clinically definitive skeletal outcomes were unavailable in the included trials. Incident fractures, particularly new vertebral fractures, provide a more direct measure of clinical benefit than changes in BMD. However, none of the included RCTs reported fracture incidence, and BMD was therefore the principal available surrogate measure of skeletal efficacy. This limitation is particularly relevant in diabetic osteoporosis because fracture risk may remain elevated despite normal or relatively preserved BMD. In addition, no eligible trial reported trabecular bone score or other bone microstructural parameters suitable for quantitative synthesis. Bone turnover markers should therefore be interpreted primarily as pharmacodynamic indicators rather than direct evidence of fracture prevention. Consequently, the observed improvements in BMD cannot be assumed to translate into reduced fracture risk. Future trials should prioritize incident fractures, particularly new vertebral fractures, and incorporate trabecular bone score or other measures of bone microarchitecture where feasible.

Fourth, TER was a composite outcome without a standardized definition across trials. Among the 21 RCTs reporting TER, the criteria used to define treatment response varied considerably. Some studies integrated changes in BMD, pain VAS scores, and TCM syndrome scores, whereas others relied primarily on BMD improvement or self reported symptoms. Because TER incorporated different response criteria and clinical components across studies, this variability may have influenced pooled TER estimates and limited their comparability with standardized clinical outcomes. Therefore, findings for TER should be interpreted with caution and should not be considered equivalent to a standardized clinical endpoint. Future trials should use clearly defined and standardized response criteria.

Fifth, other methodological limitations should be acknowledged. The treatment durations across included studies varied widely (1 month–1 year), with short-duration studies unable to reflect the true cumulative effect on bone mass. There were also differences in CCPPs dosages and treatment courses. Furthermore, all included studies were conducted in Chinese populations and published in Chinese, which may limit the external validity and generalizability of our findings to other ethnic groups, healthcare systems, and clinical practice settings. Therefore, caution should be exercised when applying these results beyond the studied populations. Future international and multicenter studies are warranted to evaluate the effectiveness and applicability of CCPPs in broader clinical contexts.

Overall, this NMA provides comparative evidence on the potential adjunctive value of oral CCPPs for DOP treatment. However, the reliability and clinical applicability of the findings are limited by the methodological quality of the included studies, the absence of fracture outcomes and bone quality measures, and the uncertainty of several network estimates. Clinicians should therefore interpret these findings cautiously when considering individualized treatment strategies. Future rigorously designed trials with clinically relevant skeletal outcomes and longer follow up are needed to confirm the comparative efficacy and safety of CCPPs in DOP.

## 5 Conclusion

The findings of this network meta analysis suggest that commercial Chinese polyherbal preparations (CCPPs) may provide additional clinical benefits when used as adjunctive therapies to biomedicine in patients with diabetic osteoporosis (DOP). Compared with biomedicine alone, CCPPs combined with biomedicine showed potential benefits in improving total effective rate (TER), bone mineral density at multiple sites, and bone turnover markers. Regarding specific treatment options, GSK had the highest SUCRA ranking for TER, while XLGB, JTG and GSK showed potential benefits for improving bone mineral density. The different regimens also showed distinct patterns across biochemical outcomes. JTG ranked highest for osteocalcin, XLGB ranked highest for calcium and phosphorus, LWDHW ranked highest for fasting blood glucose and GL had the lowest risk of adverse events. However, these rankings should be interpreted cautiously, particularly because the certainty of evidence for many comparisons was low or very low. They should therefore be considered exploratory rather than definitive evidence of comparative superiority. Selection of CCPPs should consider the magnitude and precision of treatment effects, certainty of evidence, patient characteristics, and background therapy rather than SUCRA rankings alone. Future research should include multilingual literature, prioritize high quality RCTs, cover a broader range of interventions, integrate long term follow up, and systematically assess adverse events. Further rigorous studies are needed to confirm the comparative efficacy and safety of individual CCPP regimens in patients with DOP.

## Data Availability

The original contributions presented in the study are included in the article/Supplementary Material, further inquiries can be directed to the corresponding author.
