# Supplementary material for: The efficacy and safety of combining different commercial Chinese polyherbal preparations with conventional Western drugs in the treatment of diabetic osteoporosis: a network meta-analysis
Source: Front Pharmacol. 2026 Oct 21;17:1880450. doi: 10.3389/fphar.2026.1880450 (PMC13731222; doi:10.3389/fphar.2026.1880450)
Supplement: Supplementary file 1 [file Supplementaryfile1.docx]

**Supplementary Appendix**

**The efficacy and safety of combining different commercial Chinese polyherbal preparations with conventional Western drugs in the treatment of diabetic osteoporosis: A network meta-analysis**

**Table of contents**

[**Appendix 1: Search strategy**	2](#_Toc242291148)

[**Appendix 2: Characteristics of included studies**	4](#_Toc242291149)

[**Appendix 3: List of data extracted from the included randomized clinical trials**	12](#_Toc242291150)

[**Appendix 4: Risk of bias of randomized clinical trials**	13](#_Toc242291151)

[**Appendix 5: Evaluation of inconsistency and heterogeneity**	16](#_Toc242291152)

[**Appendix 6: SUCRA and cumulative probability plots**	17](#_Toc242291153)

[**Appendix 7: League table of summary estimates for different Chinese patent medicines combined with conventional Western medicine in treating diabetic osteoporosis, derived from network meta-analysis of 34 trials.**	28](#_Toc242291154)

[**Appendix 8: CINeMA Assessment**	32](#_Toc242291155)

[**Appendix 9: Funnel plots**	63](#_Toc242291156)

**Appendix 1: Search strategy**

**Table S1.1** Search strategy

| PubMed | ("Diabetes Mellitus, Type 2"[MeSH] OR "Diabetes Mellitus, Type 2"[TIAB] OR "T2DM"[TIAB] OR "Type 2 Diabetes"[TIAB]) AND ("Osteoporosis"[MeSH] OR "Osteoporosis"[TIAB] OR "Bone Diseases, Metabolic"[MeSH] OR "Bone Resorption"[MeSH]) AND ("Drugs, Chinese Herbal"[MeSH] OR "Medicine, Chinese Traditional"[MeSH] OR "Chinese Herbal Drugs"[TIAB] OR "Chinese Patent Medicine"[TIAB] OR "Chinese Herbal Medicine"[TIAB] OR "Zhong Cheng Yao"[TIAB]) AND (randomized controlled trial[Publication Type] OR controlled clinical trial[Publication Type] OR randomized[TIAB] OR placebo[TIAB] OR "clinical trial"[TIAB]) |
| --- | --- |
| Web of Science | TS=((("Type 2 diabetes" OR "Diabetes Mellitus, Type 2" OR T2DM OR "type II diabetes" OR T2D) AND (Osteoporosis OR "Bone Resorption" OR "Bone Diseases, Metabolic")) AND ("Chinese patent medicine" OR "Chinese herbal drugs" OR "Chinese herbal medicine" OR "Oral Chinese patent" OR "proprietary Chinese medicine" OR "Zhong Cheng Yao")) AND TS=(randomized controlled trial OR controlled clinical trial OR RCT OR randomized OR placebo OR randomly) |
| Cochrane | 1 MeSH descriptor: [Diabetes Mellitus, Type 2] explode all trees  2 ("Type 2 diabetes" or T2DM or "type II diabetes" or NIDDM):ti,ab,kw  3 1 or 2  4 MeSH descriptor: [Osteoporosis] explode all trees  5 MeSH descriptor: [Bone Diseases, Metabolic] explode all trees  6 (osteoporosis or "bone resorption" or osteopenia or "low bone density"):ti,ab,kw  7 4 or 5 or 6  8 3 and 7  9 MeSH descriptor: [Drugs, Chinese Herbal] explode all trees  10 ("chinese patent medicine" or "chinese herbal drugs" or "proprietary chinese medicine" or "zhong cheng yao" or "traditional chinese medicine"):ti,ab,kw  11 9 or 10  12 8 and 11  13 (randomized controlled trial or controlled clinical trial or randomized or placebo):ti,ab,kw  14 12 and 13 |
| Embase | 1 exp *non insulin dependent diabetes mellitus/  2 (non insulin dependent diabetes or type 2 diabetes or type II diabetes or T2DM or T2D).ti,ab,kw.  3 or/1-2  4 exp *osteoporosis/  5 exp metabolic bone disease/  6 (osteoporosis or bone resorption or osteopenia or low bone density or bone loss).ti,ab,kw.  7 or/4-6  8 exp *Chinese medicine/  9 exp *Chinese patent drug/  10 exp *Chinese herbal drug/  11 (chinese patent medicine or chinese herbal drugs or proprietary chinese medicine or traditional chinese medicine or zhong cheng yao or oral chinese patent).ti,ab,kw.  12 or/8-11  13 3 and 7  14 12 and 13  15 randomized controlled trial/ or "randomized controlled trial".pt. or controlled clinical trial/ or randomized.ti,ab. or placebo.ti,ab. or randomly.ti,ab. or rct.ti,ab.  16 14 and 15 |
| CNKI、 VIP、Wanfang and CBM | SU=(type 2 diabetes mellitus + diabetes mellitus type 2 + T2DM + non-insulin-dependent diabetes mellitus) AND SU=(osteoporosis + osteopenia + bone loss + metabolic bone disease) AND SU=(Chinese patent medicine + Chinese herbal medicine + herbal medicine + Chinese herbal compound) AND SU=(randomized controlled trial + randomized + placebo + random allocation) |

**Appendix 2: Characteristics of included studies**

**Table S2.1:** Baseline of characteristics of included studies

| **Study** | **Country and region** | **Design** | **Number of participants** | **Age (mean ± SD), years** | **Sex(men/women)** | **BMI** | **Disease duration** | **Dose and frequency** | **therapy duration** | **Outcome indicator** |
| --- | --- | --- | --- | --- | --- | --- | --- | --- | --- | --- |
| S1. Elias Abula | China | RCT | 69 | 62.18±3.65 | 29/40 | 25.09±2.12 | 3.02±0.73 | Xianling Gubao Capsules(0.5g/cap) 3 caps,BID×6w | 6w | ①②③④⑤⑪ |
| 2020 |  |  | 69 | 62.04±3.87 | 28/41 | 25.17±2.03 | 2.89±0.62 | Zoledronic acid Injection(5ml:4mg) 4mg,iv.gtt,qy |  |  |
| S2. Li hui | China | RCT | 68 | 68.41±6.27 | 33/35 | 24.42±3.52 | 7.48±1.36 | Xianling Gubao Capsules(0.5g/cap) 3 caps,BID×6w | 6w | ①②③⑪ |
| 2021 |  |  | 68 | 68.36±6.32 | 31/37 | 24.35±3.46 | 7.43±1.32 | Alendronate Sodium Tablets 70mg,QW×6w Metformin Hydrochloride Tablets 0.5g,BID×6w |  |  |
| S3. Zhang ruijuan | China | RCT | 150 | 62.09±8.25 | 60/90 | - | 5.99±1.75 | Xianling Gubao Capsules(0.5g/cap) 3 caps,BID×12w | 12w | ①②③⑪ |
| 2024 |  |  | 150 | 61.95±8.48 | 62/88 | - | 6.15±1.56 | Alendronate Sodium Tablets 70mg,QW×12w |  |  |
| S4. Lin detao | China | RCT | 39 | 68.7±6.0 | 15/24 | - | - | Xianling Gubao Capsules(0.5g/cap) 3 caps,BID×12w | 12w | ①③⑤ |
| 2017 |  |  | 39 | 69.2±6.4 | 17/22 | - | - | Alendronate Sodium Tablets 70mg,QW×12w |  |  |
| S5. Zeng li | China | RCT | 100 | 59.80±9.39 | 48/52 | 24.38±2.56 | - | Xianling Gubao Capsules(0.5g/cap) 3 caps,BID×12w | 12w | ①⑥⑧⑨⑪ |
| 2024 |  |  | 100 | 58.37±11.08 | 47/53 | 23.17±2.46 | - | Calcitriol Soft Capsules(0.25ug/cap) 1 cap,QD×12w |  |  |
| S6. Yang haojie | China | RCT | 46 | 63.28±1.21 | 29/17 | - | 4.35±0.36 | Xianling Gubao Capsules(0.5g/cap) 3 caps,BID×12w | 12w | ②③④⑤ |
| 2025 |  |  | 49 | 63.26±1.18 | 30/19 | - | 4.32±0.43 | Alendronate Sodium Tablets 70mg,QW×12w |  |  |
| S7. Zhou yajing | China | RCT | 53 | 61.8±4.7 | 25/28 | - | 7.63±2.45 | Jintian Ge Capsules(0.4g/cap) 3 caps,TID×48w | 48w | ①③④⑥⑦⑩ |
| 2023 |  |  | 52 | 62.2±4.7 | 27/25 | - | 7.86±2.51 | Alfacalcidol Soft Capsules(0.25ug/cap) 1 cap,QD×48w |  |  |
| S8. Pan ying | China | RCT | 70 | 67.25±5.43 | - | - | 7.19±1.55 | Jintian Ge Capsules(0.4g/cap) 3 caps,TID×24w | 24w | ②③④⑤⑥⑦⑧⑨⑩⑪ |
| 2016 |  |  | 70 | 67.19±5.40 | - | - | 7.14±1.53 | Zoledronic acid Injection(100ml:5mg) 5mg,iv.gtt,qm×24w |  |  |
| S9. Dai jiale | China | RCT | 80 | 68.26±5.79 | - | - | 7.42±1.98 | Jintian Ge Capsules(0.4g/cap) 3 caps,TID×24w | 24w | ②③④⑤⑥⑦⑧⑨⑪ |
| 2018 |  |  | 80 | 67.33±6.35 | - | - | 7.36±2.31 | Zoledronic acid Injection(100ml:5mg) 5mg,iv.gtt,qm×24w |  |  |
| S10. Yang zhenyu | China | RCT | 47 | 60.77±5.09 | 21/26 | - | 6.05±0.62 | Jintian Ge Capsules(0.4g/cap) 3 caps,TID×12w | 12w | ①③④⑥⑦⑪ |
| 2022 |  |  | 47 | 61.39±6.12 | 22/25 | - | 5.82±0.57 | Alendronate Sodium Tablets 70mg,QW×12w |  |  |
| S11. Yuan ning | China | RCT | 100 | 62.41±7.02 | 59/41 | 23.54±2.61 | 5.43±4.08 | Jintian Ge Capsules(0.4g/cap) 3 caps,TID×24w | 24w | ①②⑥⑩ |
| 2018 |  |  | 100 | 61.74±6.34 | 61/39 | 23.04±2.40 | 5.31±3.42 | Alendronate Sodium Tablets 10mg,QW×24w |  |  |
| S12. Yan jingsong | China | RCT | 75 | - | - | - | - | Jintian Ge Capsules(0.4g/cap) 3 caps,TID×12w | 12w | ⑦⑧⑨⑪ |
| 2015 |  |  | 75 | - | - | - | - | Calcium Carbonate and Vitamin D3 Tablets 0.6g,BID×12w |  |  |
| S13. Zhang zhiliang | China | RCT | 45 | 61.98±4.76 | 20/25 | - | 8.12±2.61 | Jintian Ge Capsules(0.4g/cap) 3 caps,TID×48w | 48w | ①②③④⑥⑦⑧⑨⑩⑪ |
| 2018 |  |  | 45 | 62.85±4.86 | 22/23 | - | 8.83±2.75 | Alfacalcidol Soft Capsules(0.25ug/cap) 1 cap,QD×48w |  |  |
| S14. Gao mingsong | China | RCT | 48 | 66.41±6.08 | 23/25 | - | - | Tangmai Kang Granules(5g/bag) 1bag,TID×26w | 26w | ①②③⑥ |
| 2012 |  |  | 48 | 65.62±5.41 | 24/24 | - | - | Alendronate Sodium Tablets 10mg,QW×26w |  |  |
| S15. Yu bing | China | RCT | 52 | 65.34±1.58 | 29/23 | - | - | Tangmai Kang Granules(5g/bag) 1bag,BID×26w | 52w | ①②③⑥⑦⑧⑨ |
| 2014 |  |  | 52 | 67.57±2.09 | 25/27 | - | - | Alendronate Sodium Tablets 70mg,QW×52w |  |  |
| S16. Zhang jingjing | China | RCT | 30 | 54.02±5.15 | 12 18 | - | 10.44±2.24 | Tangmai Kang Granules(5g/bag) 1bag,TID×24w | 24w | ②⑩⑪ |
| 2015 |  |  | 30 | 54.02±5.15 | 14/16 | - | 10.44±2.24 | Alendronate Sodium Tablets 10mg,QW×24w |  |  |
| S17. Gao mingsong | China | RCT | 32 | 64.31±6.86 | 16/16 | - | 8.24±6.35 | Tangmai Kang Granules(5g/bag) 1bag,TID×20w | 20w | ①② |
| 2009 |  |  | 30 | 63.13±5.89 | 14/16 | - | 7.75±6.08 | Alendronate Sodium Tablets 10mg,QW×20w |  |  |
| S18. Gao mingsong | China | RCT | 38 | 63.23±6.64 | 19/19 | - | - | Tangmai Kang Granules(5g/bag) 1bag,TID×26w | 26w | ①②③ |
| 2008 |  |  | 35 | 63.15±5.87 | 17/18 | - | - | Alendronate Sodium Tablets 10mg,QW×26w |  |  |
| S19. Wang yang | China | RCT | 24 | 66.00±6.06 | - | - | 3.27±0.44 | Gushu Kang Capsules(0.32g/cap) 4 caps,BID×24w | 24w | ②③⑩ |
| 2025 |  |  | 24 | 68.67±9.84 | - | - | 3.42±0.37 | Alendronate Sodium Tablets 70mg,QW×24w |  |  |
| S20. Shuai lin | China | RCT | 35 | - | 19/16 | - | - | Gushu Kang Capsules(0.32g/cap) 4 caps,BID×24w | 24w | ①②③⑦⑧⑨ |
| 2018 |  |  | 35 | - | 17/18 | - | - | Calcitriol Soft Capsules(0.25ug/cap) 1 cap,QD×24w |  |  |
| S21. Li dan | China | RCT | 48 | 67.95±7.02 | 22/26 | 22.9±2.49 | 5.08±1.05 | Liuwei Dihuang Pills 8pills,TID×12w | 12w | ②⑩⑪ |
| 2023 |  |  | 47 | 68±6.56 | 22/25 | 22.95±2.56 | 5.03±1.02 | Calcitriol Soft Capsules(0.25ug/cap) 1 cap,BID×12w |  |  |
| S22. An juan | China | RCT | 30 | 55.6±5.4 | 12 18 | - | 4.3±2.5 | Liuwei Dihuang Pills 8pills,TID×12w | 12w | ①⑥⑩⑪ |
| 2022 |  |  | 30 | 56.0±5.1 | 13/17 | - | 5.1±2.9 | Alendronate Sodium Tablets 70mg,QW×12w |  |  |
| S23. Zhang yuhong | China | RCT | 40 | 72.83±2.06 | 24/16 | - | 3.42±0.18 | Liuwei Dihuang Pills 8pills,TID×24w Sitagliptin 100mg,QD×24w | 24w | ⑥⑩ |
| 2019 |  |  | 40 | 72.64±2.13 | 23/17 | - | 3.31±0.26 | Calcium Carbonate and Vitamin D3 Tablets 0.6g,BID×24w Sitagliptin 100mg,QD×24w |  |  |
| S24. Jiang minghui | China | RCT | 30 | 63.23±7.77 | 12 18 | - | - | Liuwei Dihuang Pills 8pills,TID×12w | 12w | ①⑥⑧⑩⑪ |
| 2025 |  |  | 30 | 64.33±7.72 | 14/16 | - | - | Calcitriol Soft Capsules(0.25ug/cap) 1 cap,QD×12w |  |  |
| S25. Wang xiaowei | China | RCT | 56 | 50.9±2.7 | - | 24.3±1.8 | - | Guli Capsules 0.9g,TID×24w | 24w | ①③⑥⑦⑪ |
| 2022 |  |  | 57 | 50.2±2.1 | - | 24.2±1.7 | - | Alfacalcidol Soft Capsules(0.25ug/cap) 1 cap,QD×24w |  |  |
| S26. Yang hua | China | RCT | 42 | 50.36±2.25 | - | 23.96±1.68 | - | Guli Capsules 0.9g,TID×12w | 12w | ①③⑥⑦⑪ |
| 2023 |  |  | 42 | 51.48±2.64 | - | 24.21±1.79 | - | Alfacalcidol Soft Capsules(0.25ug/cap) 0.5-1ug,QD×12w |  |  |
| S27. Li yu | China | RCT | 47 | 50.04±2.48 | - | 23.97±1.42 | - | Guli Capsules 0.9g,TID×24w | 24w | ①②③⑦⑧⑯ |
| 2024 |  |  | 47 | 50.31±2.60 | - | 24.12±1.59 | - | Calcitriol Soft Capsules(0.25ug/cap) 1 cap,QD×24w |  |  |
| S28. Li baoxin | China | RCT | 55 | 65.42±2.27 | 30/25 | - | - | Gushu Kang Granules 10g,BID×24w | 24w | ①②③⑥⑦⑪ |
| 2015 |  |  | 50 | 66.29±2.09 | 24/26 | - | - | Calcium Carbonate and Vitamin D3 Tablets 600mg,QD×24w |  |  |
| S29. Xu zhiqiang | China | RCT | 40 | 60.21±2.35 | 22/18 | - | 4.13±1.62 | Gushu Kang Granules 1bag,TID×24w | 24w | ③ |
| 2013 |  |  | 40 | 61.27±1.76 | 21/19 | - | 3.63±1.69 | Calcium Supplement with Vitamin D chewable Tablets 1 tab,QD×24w |  |  |
| S30. You yuqi | China | RCT | 60 | - | 32/28 | - | - | Xianling Gubao Capsules(0.5g/cap) 2 caps,TID×24w | 24w | ②③ |
| 2014 |  |  | 60 | - | 27/33 | - | - | Calcium Carbonate and Vitamin D3 Tablets 1 tab,QD×24w |  |  |
| S31. Liu junying | China | RCT | 35 | 53.5±4.0 | 21/14 | - | 6.25±3.37 | Tangmai Kang Granules(5g/bag) 1bag,TID×24w | 24w | ①② |
| 2014 |  |  | 35 | 54.7±3.5 | 19/16 | - | 7.43±4.52 | Alendronate Sodium Tablets 10mg,QW×24w |  |  |
| S32. Du jianru | China | RCT | 78 | - | 19/20 | - | - | Jintian Ge Capsules(0.4g/cap) 3 caps,TID×24w | 24w | ①② |
| 2014 |  |  | 78 | - | 18/21 | - | - | Alendronate Sodium Tablets 70mg,QW×24w |  |  |
| S33. Du jianru | China | RCT | 34 | - | - | - | - | Jintian Ge Capsules(0.4g/cap) 3 caps,TID×24w | 24w | ②③ |
| 2015 |  |  | 34 | - | - | - | - | Alendronate Sodium Tablets 70mg,QW×24w |  |  |
| S34. Wang limin | China | RCT | 68 | 65.8±5.8 | 35/33 | - | - | Jintian Ge Capsules(0.4g/cap) 3 caps,TID×8w | 8w | ①②⑪ |
| 2018 |  |  | 68 | 63.2±6.4 | 38/30 | - | - | Alendronate Sodium Tablets 70mg,QW×8w |  |  |

①Total effective rate; ②Lumbar spine bone mineral density; ③Femoral neck bone mineral density; ④Femoral trochanter bone mineral density; ⑤Ward's triangle bone mineral density; ⑥Osteocalcin; ⑦Alkaline phosphatase; ⑧Calcium; ⑨Phosphorus; ⑩Fasting blood glucose; ⑪Adverse events.

**Table S2.2:** Characteristics and Formulations of Included Chinese Proprietary Medicines

| Medicine | Therapeutic Action | Indication | Composition |
| --- | --- | --- | --- |
| Xianling Gubao Capsules | Nourish the liver and kidney, activate blood and promote meridian circulation, strengthen tendons and bones. | For osteoporosis, fractures, osteoarthritis, osteonecrosis, etc. | Epimedium, Dipsacus, Salvia miltiorrhiza, Anemarrhena, Psoralea, Rehmannia. |
| Jintian Ge Capsules | Exhibits bone-strengthening effects. | For the improvement of symptoms such as low back pain, soreness and weakness in the lumbar and knee regions, weakness of the lower limbs, and walking difficulty. | Artificial Tiger Bone Powder. |
| Tangmai Kang Granules | Nourish yin and clear heat, activate blood and dispel stasis, tonify qi and strengthen the kidneys. | Used for fatigue and weakness caused by diabetes with both qi and yin deficiency and blood stasis, manifesting as shortness of breath, reluctance to speak, spontaneous sweating, night sweats, heat in the five palms, thirst with a preference for drinking, chest oppression and pain, numbness or tingling in the limbs, constipation, a red tongue with little moisture, a large tongue, a thin or peeled tongue coating, or a dark tongue with stasis spots, and a pulse that is wiry and thin, or fine and rapid, or deep and rough, along with symptoms of type 2 diabetes complications exhibiting the above patterns. | Astragalus membranaceus, Rehmannia glutinosa, Paeonia veitchii, Salvia miltiorrhiza, Achyranthes bidentata, Ophiopogon japonicus, Polygonatum sibiricum, Epimedium brevicornu, Pueraria lobata. |
| Gushu Kang Capsules/Granules | Tonify the kidneys and replenish qi, activate blood and strengthen bones. | Used for primary osteoporosis caused by kidney deficiency and insufficiency of qi and blood, with symptoms including lower back pain, soreness in the lower back and knees, weakness in the lower limbs, difficulty walking, fatigue, dizziness, a tongue that is slightly red or pale, and a pulse that is either weak or thin. | Epimedium, Cooked Rehmannia, Drynaria, Astragalus, Salvia, Wood Ear Mushroom, Cucumber Seed. |
| Liuwei Dihuang Pills | Nourish yin and tonify the kidneys. | Used for kidney yin deficiency, with symptoms including dizziness, tinnitus, soreness in the lower back and knees, afternoon fever, night sweats, and seminal emissions. | Cooked Rehmannia, Wolfberry fruit (Goji), Chinese Yam, Peony root bark, Poria, Alisma. |
| Guli Capsules | Strengthen the muscles and bones, expel wind-damp, activate blood and dispel stasis, unblock channels and relieve pain. | Primarily used for low back and leg soreness, limb numbness, and osteoporosis caused by wind, cold, and dampness obstructing the meridians. | Epimedium, Cistanches, Achyranthes, Radix clematis, Papaya, Turmeric, Psoralea, Codonopsis, Kudzu. |

**Table S2.3:** Manufacturer Details and Regulatory Information of Included Medicines

| Medicine | Manufacturer | Specification | Approval Number |
| --- | --- | --- | --- |
| Xianling Gubao Capsules | Guizhou Tongjitang Pharmaceutical Co., Ltd. | 0.5g/cap | Z20025337 |
| Jintian Ge Capsules | Jinhua Enterprise (Group) Co., Ltd. | 0.5g/cap | Z20030080 |
| Tangmai Kang Granules | Chengdu Zhonghui Pharmaceutical Co., Ltd. | 5 g/bag | Z10970026 |
| Gushu Kang Capsules | Liaoning Kangchen Pharmaceutical Co., Ltd. | 0.32g/cap | Z20060237 |
| Gushu Kang Granules | Liaoning Kangchen Pharmaceutical Co., Ltd. | 10g/bag | Z20003255 |
| Liuwei Dihuang Pills | Henan Wanxi Pharmaceutical Co., Ltd. |  | Z401022128 |
|  | Shandong Kongshengtang Pharmaceutical Co., Ltd. |  | Z10983129 |
|  | Jiuzhitang Co., Ltd. |  | Z43020145 |
| Guli Capsules | Guizhou Bailing Enterprise Group Pharmaceutical Co., Ltd. | 0.3g/cap | Z20027661 |

**Appendix 3: List of data extracted from the included randomized clinical trials**

| Data category | List of variables |
| --- | --- |
| Study | Primary author, year of publication, duration of study, total number of patients in each group |
| Patients | Age, sex, BMI, disease duration |
| Interventions | Drug class, dose and duration of the primary intervention and strategies used for implementing them |
|  |  |
| Efficacy outcome | BMD, OC, ALP, Ca, P, FPG |
|  |  |
| Adverse events | Various adverse events reported in included trials |

**Appendix 4: Risk of bias of randomized clinical trials**

**Figure S4:** Overall risk of bias presented as percentage of each risk of bias item across all included studies. Green = Low risk, Red = High risk, Yellow = Some concerns.

**Table S4:** Study level risk of bias assessment using Cochrane risk of bias tool 2.0 for assessing risk of bias of randomized clinical trials.

| Unique ID | Randomization process | Deviations from intended interventions | Missing outcome data | Measurement of the outcome | Selection of the reported result | Over all |
| --- | --- | --- | --- | --- | --- | --- |
| S1: Elias Abula2020 | Low | some concerns | Low | Low | Low | some concerns |
| S2: Li hui2021 | Low | some concerns | Low | Low | Low | some concerns |
| S3: Zhang ruijuan2024 | Low | some concerns | Low | Low | Low | some concerns |
| S4: Lin detao2017 | Low | some concerns | Low | Low | Low | some concerns |
| S5: Zeng li2024 | Low | some concerns | Low | Low | Low | some concerns |
| S6: Yang haojie2025 | Low | some concerns | Low | Low | Low | some concerns |
| S7: Zhou yajing2023 | Low | some concerns | Low | Low | Low | some concerns |
| S8: Pan ying2016 | Low | some concerns | Low | Low | Low | some concerns |
| S9: Dai jiale2018 | Low | some concerns | Low | Low | Low | some concerns |
| S10: Yang zhenyu2022 | Low | some concerns | Low | Low | Low | some concerns |
| S11: Yuan ning2018 | Low | some concerns | Low | Low | Low | some concerns |
| S12: Yan jingsong2015 | some concerns | some concerns | Low | Low | Low | high |
| S13: Zhang zhiliang2018 | Low | some concerns | Low | Low | Low | some concerns |
| S14: Gao mingsong2012 | some concerns | some concerns | Low | Low | Low | high |
| S15: Yu bing2014 | Low | some concerns | Low | Low | Low | some concerns |
| S16: Zhang jingjing2015 | some concerns | some concerns | Low | Low | Low | high |
| S17: Gao mingsong2009 | some concerns | some concerns | Low | Low | Low | high |
| S18: Gao mingsong2008 | some concerns | some concerns | Low | Low | Low | high |
| S19: Wang yang2025 | Low | some concerns | Low | Low | Low | some concerns |
| S20: Shuai lin2018 | Low | some concerns | Low | Low | Low | some concerns |
| S21: Li dan2023 | Low | some concerns | Low | Low | Low | some concerns |
| S22: An juan2022 | some concerns | some concerns | Low | Low | Low | high |
| S23: Zhang yuhong2019 | Low | some concerns | Low | Low | Low | some concerns |
| S24: Jiang minghui2025 | Low | some concerns | Low | Low | Low | some concerns |
| S25: Wang xiaowei2022 | Low | some concerns | Low | Low | Low | some concerns |
| S26: Yang hua2023 | Low | some concerns | Low | Low | Low | some concerns |
| S27: Li yu2024 | Low | some concerns | Low | Low | Low | some concerns |
| S28: Li baoxin2015 | some concerns | some concerns | Low | Low | Low | high |
| S29: Xu zhiqiang2013 | some concerns | some concerns | Low | Low | Low | high |
| S30: You yuqi2014 | Low | some concerns | Low | Low | Low | some concerns |
| S31: Liu junying2014 | Low | some concerns | Low | Low | Low | some concerns |
| S32: Du jianru2014 | some concerns | some concerns | Low | Low | Low | high |
| S33: Du jianru2015 | Low | some concerns | Low | Low | Low | some concerns |
| S34: Wang limin2018 | Low | some concerns | Low | Low | Low | some concerns |

**Appendix 5: Evaluation of inconsistency and heterogeneity**

**Table S5:** Global consistency and Heterogeneity score

| Clinical outcome | Chi square | P value | τ2 network |
| --- | --- | --- | --- |
| Total effective rate | 11.33 | 0.0008 | 8.827e-16 |
| Lumbar spine bone mineral density | 17.20 | 0.0000 | 0.0007 |
| Femoral neck bone mineral density | 0.31 | 0.5796 | 0.0172 |
| Femoral greater trochanter bone mineral density | 0.54 | 0.4622 | 0.0026 |
| Ward’s triangle bone mineral density | 6.29 | 0.0122 | 0.0002 |
| Osteocalcin | 5.47 | 0.0194 | 0.4953 |
| ALP | 0.51 | 0.4738 | 4.3240 |
| Ca | 0.16 | 0.6865 | 0.0042 |
| P | 0.13 | 0.7185 | 0.00002 |
| FBG | 8.88 | 0.0029 | 0.4247 |
| Adverse events | 2.60 | 0.1066 | 5.696e-19 |

**Appendix 6: SUCRA and cumulative probability plots**

**Figure S6.1:** Cumulative ranking curve plots of lumbar spine bone mineral density for different Chinese patent medicines combined with conventional Western medicine in treating diabetic osteoporosis within the scope of the network. The larger the area under the curve, the more effective the treatment.

**Table S6.1:** SUCRA rankings of lumbar spine bone mineral density for various Chinese patent medicines combined with conventional Western medicine in treating diabetic osteoporosis.

| **Treatment** | **SUCRA** | **Prbest** | **MeanRank** |
| --- | --- | --- | --- |
| XLGB | 80.7 | 7.9 | 2.2 |
| JTG | 52.5 | 0.1 | 3.9 |
| TMK | 39.5 | 0 | 4.6 |
| GSK | 24.8 | 0 | 5.5 |
| LWDHW | 54.6 | 2 | 3.7 |
| GL | 97.6 | 90.1 | 1.1 |
| biomedicine | 0.3 | 0 | 7 |

Abbreviations: SUCRA, surface under the cumulative ranking curve;

XLGB, Xianling Gubao Capsules;

JTG, Jintian Ge Capsules;

TMK, Tangmai Kang Granules;

GSK, Gushu Kang Capsules/Granules;

LWDHW, Liuwei Dihuang Pills;

GL, Guli Capsules.

**Figure S6.2:** Cumulative ranking curve plots of femoral neck bone mineral density for different Chinese patent medicines combined with conventional Western medicine in treating diabetic osteoporosis within the scope of the network. The larger the area under the curve, the more effective the treatment.

**Table S6.2:** SUCRA rankings of femoral neck bone mineral density for various Chinese patent medicines combined with conventional Western medicine in treating diabetic osteoporosis.

| **Treatment** | **SUCRA** | **Prbest** | **MeanRank** |
| --- | --- | --- | --- |
| XLGB | 72.2 | 27.9 | 2.4 |
| JTG | 40 | 3.5 | 4 |
| TMK | 45.8 | 10 | 3.7 |
| GSK | 81.8 | 49.2 | 1.9 |
| LWDHW | 43.4 | 9.4 | 3.8 |
| GL | 16.9 | 0 | 5.2 |
| biomedicine | 72.2 | 27.9 | 2.4 |

Abbreviations: SUCRA, surface under the cumulative ranking curve;

XLGB, Xianling Gubao Capsules;

JTG, Jintian Ge Capsules;

TMK, Tangmai Kang Granules;

GSK, Gushu Kang Capsules/Granules;

LWDHW, Liuwei Dihuang Pills;

GL, Guli Capsules.

**Figure S6.3:** Cumulative ranking curve plots of femoral greater trochanter bone mineral density for different Chinese patent medicines combined with conventional Western medicine in treating diabetic osteoporosis within the scope of the network. The larger the area under the curve, the more effective the treatment.

**Table S6.3:** SUCRA rankings of femoral greater trochanter bone mineral density for various Chinese patent medicines combined with conventional Western medicine in treating diabetic osteoporosis.

| **Treatment** | **SUCRA** | **Prbest** | **MeanRank** |
| --- | --- | --- | --- |
| XLGB | 56.7 | 23.5 | 2.3 |
| JTG | 86.5 | 65.2 | 1.4 |
| GSK | 43.8 | 11.3 | 2.7 |
| biomedicine | 12.9 | 0 | 3.6 |

Abbreviations: SUCRA, surface under the cumulative ranking curve;

XLGB, Xianling Gubao Capsules;

JTG, Jintian Ge Capsules;

GSK, Gushu Kang Capsules/Granules.

**Figure S6.4:** Cumulative ranking curve plots of bone mineral density of Ward’s triangle for different Chinese patent medicines combined with conventional Western medicine in treating diabetic osteoporosis within the scope of the network. The larger the area under the curve, the more effective the treatment.

**Table S6.4:** SUCRA rankings of bone mineral density of Ward’s triangle for various Chinese patent medicines combined with conventional Western medicine in treating diabetic osteoporosis.

| **Treatment** | **SUCRA** | **Prbest** | **MeanRank** |
| --- | --- | --- | --- |
| XLGB | 80 | 52.1 | 1.6 |
| JTG | 76.1 | 39.7 | 1.7 |
| GSK | 43.7 | 8.2 | 2.7 |
| LWDHW | 0.2 | 0 | 4 |
| biomedicine | 80 | 52.1 | 1.6 |

Abbreviations: SUCRA, surface under the cumulative ranking curve;

XLGB, Xianling Gubao Capsules;

JTG, Jintian Ge Capsules;

GSK, Gushu Kang Capsules/Granules;

LWDHW, Liuwei Dihuang Pills.

**Figure S6.5:** Cumulative ranking curve plots of osteocalcin for different Chinese patent medicines combined with conventional Western medicine in treating diabetic osteoporosis within the scope of the network. The larger the area under the curve, the more effective the treatment.

**Table S6.5:** SUCRA rankings of osteocalcin of various Chinese patent medicines combined with conventional Western medicine in treating diabetic osteoporosis.

| **Treatment** | **SUCRA** | **Prbest** | **MeanRank** |
| --- | --- | --- | --- |
| XLGB | 5.4 | 0 | 6.7 |
| JTG | 96.6 | 82.2 | 1.2 |
| TMK | 53.7 | 1.8 | 3.8 |
| GSK | 60.5 | 10.4 | 3.4 |
| LWDHW | 41.4 | 0.5 | 4.5 |
| GL | 69.3 | 5.1 | 2.8 |
| biomedicine | 23.1 | 0 | 5.6 |

Abbreviations: SUCRA, surface under the cumulative ranking curve;

XLGB, Xianling Gubao Capsules;

JTG, Jintian Ge Capsules;

TMK, Tangmai Kang Granules;

GSK, Gushu Kang Capsules/Granules;

LWDHW, Liuwei Dihuang Pills;

GL, Guli Capsules.

**Figure S6.6:** Cumulative ranking curve plots of alkaline phosphatase for different Chinese patent medicines combined with conventional Western medicine in treating diabetic osteoporosis within the scope of the network. The larger the area under the curve, the more effective the treatment.

**Table S6.6:** SUCRA rankings of alkaline phosphatase of various Chinese patent medicines combined with conventional Western medicine in treating diabetic osteoporosis.

| **Treatment** | **SUCRA** | **Prbest** | **MeanRank** |
| --- | --- | --- | --- |
| JTG | 44.5 | 9.6 | 3.2 |
| TMK | 67.2 | 51.2 | 2.3 |
| GSK | 47 | 19.3 | 3.1 |
| LWDHW | 31.3 | 7.6 | 3.7 |
| GL | 60.1 | 12.3 | 2.6 |
| biomedicine | 44.5 | 9.6 | 3.2 |

Abbreviations: SUCRA, surface under the cumulative ranking curve;

JTG, Jintian Ge Capsules;

TMK, Tangmai Kang Granules;

GSK, Gushu Kang Capsules/Granules;

LWDHW, Liuwei Dihuang Pills;

GL, Guli Capsules.

**Figure S6.7:** Cumulative ranking curve plots of serum calcium for different Chinese patent medicines combined with conventional Western medicine in treating diabetic osteoporosis within the scope of the network. The larger the area under the curve, the more effective the treatment.

**Table S6.7:** SUCRA rankings of serum calcium of various Chinese patent medicines combined with conventional Western medicine in treating diabetic osteoporosis.

| **Treatment** | **SUCRA** | **Prbest** | **MeanRank** |
| --- | --- | --- | --- |
| XLGB | 92.1 | 65 | 1.4 |
| JTG | 36.6 | 0.1 | 4.2 |
| TMK | 74.8 | 32 | 2.3 |
| GSK | 63.2 | 2.7 | 2.8 |
| LWDHW | 14.7 | 0.2 | 5.3 |
| biomedicine | 18.6 | 0 | 5.1 |

Abbreviations: SUCRA, surface under the cumulative ranking curve;

XLGB, Xianling Gubao Capsules;

JTG, Jintian Ge Capsules;

TMK, Tangmai Kang Granules;

GSK, Gushu Kang Capsules/Granules;

LWDHW, Liuwei Dihuang Pills.

**Figure S6.8:** Cumulative ranking curve plots of serum phosphorus for different Chinese patent medicines combined with conventional Western medicine in treating diabetic osteoporosis within the scope of the network. The larger the area under the curve, the more effective the treatment.

**Table S6.8:** SUCRA rankings of serum phosphorus of various Chinese patent medicines combined with conventional Western medicine in treating diabetic osteoporosis.

| **Treatment** | **SUCRA** | **Prbest** | **MeanRank** |
| --- | --- | --- | --- |
| XLGB | 93.4 | 73.6 | 1.3 |
| JTG | 12.0 | 0.0 | 4.5 |
| TMK | 73.0 | 26.4 | 2.1 |
| GSK | 39.2 | 0.0 | 3.4 |
| biomedicine | 32.4 | 0.0 | 3.7 |

Abbreviations: SUCRA, surface under the cumulative ranking curve;

XLGB, Xianling Gubao Capsules;

JTG, Jintian Ge Capsules;

TMK, Tangmai Kang Granules;

GSK, Gushu Kang Capsules/Granules.

**Figure S6.9:** Cumulative ranking curve plots of fasting blood glucose for different Chinese patent medicines combined with conventional Western medicine in treating diabetic osteoporosis within the scope of the network. The larger the area under the curve, the more effective the treatment.

**Table S6.9:** SUCRA rankings of fasting blood glucose of various Chinese patent medicines combined with conventional Western medicine in treating diabetic osteoporosis.

| **Treatment** | **SUCRA** | **Prbest** | **MeanRank** |
| --- | --- | --- | --- |
| JTG | 65.8 | 18.5 | 2.4 |
| TMK | 27 | 6.4 | 3.9 |
| GSK | 48.5 | 17.6 | 3.1 |
| LWDHW | 86.1 | 57.4 | 1.6 |
| biomedicine | 22.6 | 0 | 4.1 |

Abbreviations: SUCRA, surface under the cumulative ranking curve;

JTG, Jintian Ge Capsules;

TMK, Tangmai Kang Granules;

GSK, Gushu Kang Capsules/Granules;

LWDHW, Liuwei Dihuang Pills.

**Figure S6.10:** Cumulative ranking curve plots of total effective rate for different Chinese patent medicines combined with conventional Western medicine in treating diabetic osteoporosis within the scope of the network. The larger the area under the curve, the more effective the treatment.

**Table S6.10:** SUCRA rankings of effective rates of various Chinese patent medicines combined with conventional Western medicine in treating diabetic osteoporosis.

| **Treatment** | **SUCRA** | **Prbest** | **MeanRank** |
| --- | --- | --- | --- |
| XLGB | 30.4 | 0 | 5.2 |
| JTG | 28.3 | 0 | 5.3 |
| TMK | 62.2 | 0.8 | 3.3 |
| GSK | 96.2 | 86.5 | 1.2 |
| LWDHW | 78.6 | 11.9 | 2.3 |
| GL | 54.3 | 0.8 | 3.7 |
| biomedicine | 0 | 0 | 7 |

Abbreviations: SUCRA, surface under the cumulative ranking curve;

XLGB, Xianling Gubao Capsules;

JTG, Jintian Ge Capsules;

TMK, Tangmai Kang Granules;

GSK, Gushu Kang Capsules/Granules;

LWDHW, Liuwei Dihuang Pills;

GL, Guli Capsules.

**Figure S6.11:** Cumulative ranking curve plots of adverse events for different Chinese patent medicines combined with conventional Western medicine in treating diabetic osteoporosis within the scope of the network. The larger the area under the curve, the more effective the treatment.

Adverse reactions

**Table S6.11:** SUCRA rankings of adverse events for various Chinese patent medicines combined with conventional Western medicine in treating diabetic osteoporosis.

| **Treatment** | **SUCRA** | **Prbest** | **MeanRank** |
| --- | --- | --- | --- |
| XLGB | 58.2 | 5.1 | 3.1 |
| JTG | 32.1 | 1.1 | 4.4 |
| TMK | 71 | 53.2 | 2.5 |
| LWDHW | 24 | 2.7 | 4.8 |
| GL | 81.1 | 37.9 | 1.9 |
| biomedicine | 33.5 | 0.1 | 4.3 |

Abbreviations: SUCRA, surface under the cumulative ranking curve;

XLGB, Xianling Gubao Capsules;

JTG, Jintian Ge Capsules;

TMK, Tangmai Kang Granules;

LWDHW, Liuwei Dihuang Pills;

GL, Guli Capsules.

**Appendix 7: League table of summary estimates for different Chinese patent medicines combined with conventional Western medicine in treating diabetic osteoporosis, derived from network meta-analysis of 34 trials.**

**Table S7.1:** Lumbar spine bone mineral density

| XLGB |  |  |  |  |  |  |
| --- | --- | --- | --- | --- | --- | --- |
| 0.04 (-0.01,0.08) | JTG |  |  |  |  |  |
| **0.05 (0.01,0.09)** | 0.01 (-0.02,0.05) | TMK |  |  |  |  |
| **0.07 (0.02,0.12)** | 0.03 (-0.02,0.08) | 0.02 (-0.03,0.06) | GSK |  |  |  |
| 0.03 (-0.04,0.11) | -0.00 (-0.07,0.06) | -0.02 (-0.08,0.05) | -0.03 (-0.11,0.04) | LWDHW |  |  |
| -0.07 (-0.16,0.03) | -0.10 (-0.19,-0.01) | -0.12 (-0.20,-0.03) | -0.13 (-0.23,-0.04) | -0.10 (-0.21,0.01) | GL |  |
| 0.11 (0.08,0.15) | 0.08 (0.05,0.10) | 0.06 (0.04,0.09) | 0.05 (0.01,0.08) | 0.08 (0.02,0.14) | 0.18 (0.09,0.27) | biomedicine |

**Table S7.2:** Femoral neck bone mineral density

| XLGB |  |  |  |  |  |
| --- | --- | --- | --- | --- | --- |
| 0.07 (-0.09,0.23) | JTG |  |  |  |  |
| 0.06 (-0.13,0.25) | -0.01 (-0.19,0.17) | TMK |  |  |  |
| -0.03 (-0.21,0.15) | -0.10 (-0.27,0.07) | -0.09 (-0.29,0.11) | GSK |  |  |
| 0.06 (-0.13,0.26) | -0.00 (-0.19,0.18) | 0.01 (-0.21,0.22) | 0.09 (-0.11,0.30) | GL |  |
| 0.11 (-0.01,0.22) | 0.04 (-0.07,0.15) | 0.05 (-0.10,0.20) | 0.14 (0.01,0.27) | 0.04 (-0.11,0.20) | biomedicine |

**Table S7.3:** Femoral greater trochanter bone mineral density

| XLGB |  |  |  |
| --- | --- | --- | --- |
| -0.04 (-0.17,0.08) | JTG |  |  |
| 0.02 (-0.14,0.18) | 0.06 (-0.06,0.18) | GSK |  |
| 0.06 (-0.06,0.18) | 0.10 (0.05,0.16) | 0.04 (-0.07,0.15) | biomedicine |

**Table S7.4:** Ward’s triangle bone mineral density

| XLGB |  |  |  |
| --- | --- | --- | --- |
| 0.00 (-0.04,0.05) | JTG |  |  |
| 0.03 (-0.03,0.09) | 0.03 (-0.03,0.08) | GSK |  |
| 0.09 (0.05,0.13) | 0.09 (0.06,0.11) | 0.06 (0.01,0.11) | biomedicine |

**Table S7.5:** Osteocalcin

| XLGB |  |  |  |  |  |  |
| --- | --- | --- | --- | --- | --- | --- |
| -**2.63 (-4.15,-1.10)** | JTG |  |  |  |  |  |
| -1.45 (-3.18,0.29) | **1.18 (0.00,2.35)** | TMK |  |  |  |  |
| -1.66 (-3.68,0.35) | 0.96 (-0.59,2.52) | -0.22 (-1.97,1.54) | GSK |  |  |  |
| -1.17 (-2.92,0.58) | **1.45 (0.26,2.65)** | 0.28 (-1.17,1.73) | 0.49 (-1.28,2.26) | LWDHW |  |  |
| **-1.82 (-3.46,-0.18)** | 0.81 (-0.22,1.83) | -0.37 (-1.69,0.94) | -0.16 (-1.82,1.50) | -0.65 (-1.98,0.68) | GL |  |
| -0.83 (-2.23,0.58) | 1.80 (1.21,2.39) | 0.62 (-0.39,1.64) | 0.84 (-0.60,2.27) | 0.35 (-0.69,1.38) | 0.99 (0.16,1.83)) | biomedicine |

**Table S7.6:** ALP

| JTG |  |  |  |  |
| --- | --- | --- | --- | --- |
| 1.01 (-3.42,5.43) | TMK |  |  |  |
| 0.07 (-3.28,3.41) | -0.94 (-5.96,4.08) | GSK |  |  |
| -0.49 (-3.39,2.41) | -1.50 (-6.23,3.23) | -0.56 (-4.30,3.18) | GL |  |
| 0.37 (-1.30,2.05) | -0.63 (-4.73,3.46) | 0.31 (-2.59,3.20) | 0.86 (-1.50,3.23) | biomedicine |

**Table S7.7:** Ca

| XLGB |  |  |  |  |  |
| --- | --- | --- | --- | --- | --- |
| **0.21 (0.05,0.38)** | JTG |  |  |  |  |
| 0.07 (-0.22,0.36) | -0.14 (-0.41,0.12) | TMK |  |  |  |
| 0.14 (-0.03,0.31) | -0.07 (-0.20,0.05) | 0.07 (-0.20,0.34) | GSK |  |  |
| **0.28 (0.08,0.48)** | 0.07 (-0.10,0.23) | 0.21 (-0.08,0.50) | 0.14 (-0.03,0.31) | LWDHW |  |
| 0.25 (0.11,0.39) | 0.04 (-0.05,0.12) | 0.18 (-0.07,0.43) | 0.11 (0.02,0.20) | -0.03 (-0.18,0.12) | biomedicine |

**Table S7.8:** P

| XLGB |  |  |  |  |
| --- | --- | --- | --- | --- |
| **0.25 (0.18,0.33)** | JTG |  |  |  |
| 0.08 (-0.18,0.34) | -0.17 (-0.42,0.08) | TMK |  |  |
| **0.22 (0.15,0.29)** | -0.03 (-0.09,0.03) | 0.14 (-0.11,0.39) | GSK |  |
| 0.23 (0.17,0.29) | -0.02 (-0.06,0.02) | 0.15 (-0.10,0.40) | 0.01 (-0.03,0.04) | biomedicine |

**Table S7.9:** FBG

| JTG |  |  |  |  |
| --- | --- | --- | --- | --- |
| -0.73 (-2.40,0.93) | TMK |  |  |  |
| -0.27 (-1.99,1.44) | 0.46 (-1.63,2.55) | GSK |  |  |
| 0.37 (-0.69,1.44) | 1.11 (-0.50,2.71) | 0.65 (-1.00,2.29) | LWDHW |  |
| -0.65 (-1.48,0.17) | 0.08 (-1.37,1.53) | -0.38 (-1.88,1.12) | -1.03 (-1.70,-0.35) | biomedicine |

**Table S7.10:** Total effective rate

| XLGB |  |  |  |  |  |  |
| --- | --- | --- | --- | --- | --- | --- |
| 1.00 (0.94,1.08) | JTG |  |  |  |  |  |
| 0.93 (0.84,1.02) | 0.92 (0.84,1.02) | TMK |  |  |  |  |
| **0.68 (0.49,0.94)** | **0.68 (0.49,0.94)** | 0.73 (0.53,1.02) | GSK |  |  |  |
| 0.87 (0.69,1.10) | 0.87 (0.69,1.09) | 0.94 (0.74,1.19) | 1.28 (0.86,1.89) | LWDHW |  |  |
| 0.94 (0.83,1.07) | 0.94 (0.83,1.06) | 1.02 (0.89,1.17) | 1.39 (0.99,1.95) | 1.09 (0.84,1.40) | GL |  |
| 1.15 (1.09,1.20) | 1.14 (1.08,1.20) | 1.24 (1.14,1.34) | 1.68 (1.22,2.32) | 1.32 (1.05,1.65) | 1.21 (1.08,1.36) | biomedicine |

**Table S7.11:** Adverse events

| XLGB |  |  |  |  |  |
| --- | --- | --- | --- | --- | --- |
| 0.72 (0.32,1.60) | JTG |  |  |  |  |
| 2.32 (0.09,61.81) | 3.24 (0.12,87.45) | TMK |  |  |  |
| 0.55 (0.12,2.39) | 0.76 (0.17,3.43) | 0.24 (0.01,7.97) | LWDHW |  |  |
| 1.83 (0.54,6.19) | 2.57 (0.74,8.93) | 0.79 (0.03,24.18) | 3.36 (0.58,19.44) | GL |  |
| 0.75 (0.44,1.28) | 1.04 (0.57,1.91) | 0.32 (0.01,8.23) | 1.37 (0.35,5.41) | 0.41 (0.14,1.21) | biomedicine |

**Appendix 8: CINeMA Assessment**

We use the CINeMA framework to evidence certainty, assessing it for each network estimate based on the following criteria:

**Within study bias:** We classified the overall risk of bias for each study as low risk of bias, the risk of bias as moderate when none of the four assessed risk of bias items were rated as high risk, and the risk of bias as high when one or both items were rated as high risk. See Appendix 4 for the bias assessment. The risk of bias for a pairwise comparison of each drug is shown in figure S10.1-10.22.

**Figure S8.1:** Risk of bias contribution by intervention group in Lumbar spine bone mineral density

**Figure S8.2:** Overall risk of bias by treatment comparison in Lumbar spine bone mineral density

**Figure S8.3:** Risk of bias contribution by intervention group in Femoral neck bone mineral density

**Figure S8.4:** Overall risk of bias by treatment comparison in Femoral neck bone mineral density

**Figure S8.5:** Risk of bias contribution by intervention group in Femoral greater trochanter bone mineral density

**Figure S8.6:** Overall risk of bias by treatment comparison in Femoral greater trochanter bone mineral density

**Figure S8.7:** Risk of bias contribution by intervention group in Ward’s triangle bone mineral density

**Figure S8.8:** Overall risk of bias by treatment comparison in Ward’s triangle bone mineral density

**Figure S8.9:** Risk of bias contribution by intervention group in Osteocalcin

**Figure S8.10:** Overall risk of bias by treatment comparison in Osteocalcin

**Figure S8.11:** Risk of bias contribution by intervention group in ALP

**Figure S8.12:** Overall risk of bias by treatment comparison in ALP

**Figure S8.13:** Risk of bias contribution by intervention group in Ca

**Figure S8.14:** Overall risk of bias by treatment comparison in Ca

**Figure S8.15:** Risk of bias contribution by intervention group in P

**Figure S8.16:** Overall risk of bias by treatment comparison in P

**Figure S8.17:** Risk of bias contribution by intervention group in FBG

**Figure S8.18:** Overall risk of bias by treatment comparison in FBG

**Figure S8.19:** Risk of bias contribution by intervention group in Effective rate

**Figure S8.20:** Overall risk of bias by treatment comparison in Effective rate

**Figure S8.21:** Risk of bias contribution by intervention group in Adverse events

**Figure S8.22:** Overall risk of bias by treatment comparison in Adverse events

**Imprecision:** We use the CINeMA website to grade the accuracy of each comparison.

**Heterogeneity:** We assessed the degree of worry by comparing clinical reasoning based on 95% confidence intervals (CIs) while applying the same clinical reasoning framework as for inaccuracy. In particular, we judged the consistency of our findings based on the confidence and prediction intervals associated with clinically important effect sizes. And we used the same thresholds of clinical significance as described above and followed the recommendations automatically provided by CINeMA (https://cinema.ispm.unibe.ch/).

**Table S8.1:** CINeMA Results of Lumbar spine bone mineral density

| Comparison | Within-study bias | Reporting bias | Indirectness | Imprecision | Heterogeneity | Incoherence | Confidence rating |
| --- | --- | --- | --- | --- | --- | --- | --- |
| GL+WMT:WMT | Some concerns | Low risk | No concerns | No concerns | No concerns | Major concerns | Moderate |
| GSK+WMT:WMT | Some concerns | Low risk | No concerns | No concerns | Major concerns | Major concerns | Low |
| JTG+WMT:WMT | Some concerns | Low risk | No concerns | No concerns | No concerns | Major concerns | Moderate |
| LWDHW+WMT:WMT | Some concerns | Low risk | No concerns | Major concerns | No concerns | Major concerns | Very low |
| TMK+WMT:WMT | Major concerns | Low risk | No concerns | No concerns | No concerns | Major concerns | Low |
| WMT:XLGB+WMT | Some concerns | Low risk | No concerns | No concerns | No concerns | Major concerns | Moderate |
| GL+WMT:GSK+WMT | Some concerns | Low risk | No concerns | No concerns | No concerns | Major concerns | Moderate |
| GL+WMT:JTG+WMT | Some concerns | Low risk | No concerns | No concerns | Major concerns | Major concerns | Low |
| GL+WMT:LWDHW+WMT | Some concerns | Low risk | No concerns | No concerns | Major concerns | Major concerns | Low |
| GL+WMT:TMK+WMT | Some concerns | Low risk | No concerns | No concerns | No concerns | Major concerns | Moderate |
| GL+WMT:XLGB+WMT | Some concerns | Low risk | No concerns | Major concerns | No concerns | Major concerns | Very low |
| GSK+WMT:JTG+WMT | Some concerns | Low risk | No concerns | Major concerns | No concerns | Major concerns | Very low |
| GSK+WMT:LWDHW+WMT | Some concerns | Low risk | No concerns | Major concerns | No concerns | Major concerns | Very low |
| GSK+WMT:TMK+WMT | Some concerns | Low risk | No concerns | Major concerns | No concerns | Major concerns | Very low |
| GSK+WMT:XLGB+WMT | Some concerns | Low risk | No concerns | No concerns | Major concerns | Major concerns | Low |
| JTG+WMT:LWDHW+WMT | Some concerns | Low risk | No concerns | Major concerns | No concerns | Major concerns | Very low |
| JTG+WMT:TMK+WMT | Some concerns | Low risk | No concerns | Major concerns | No concerns | Major concerns | Very low |
| JTG+WMT:XLGB+WMT | Some concerns | Low risk | No concerns | Major concerns | No concerns | Major concerns | Very low |
| LWDHW+WMT:TMK+WMT | Some concerns | Low risk | No concerns | Major concerns | No concerns | Major concerns | Very low |
| LWDHW+WMT:XLGB+WMT | Some concerns | Low risk | No concerns | Major concerns | No concerns | Major concerns | Very low |
| TMK+WMT:XLGB+WMT | Some concerns | Low risk | No concerns | No concerns | Major concerns | Major concerns | Low |

**Table S8.2:** CINeMA Results of Femoral neck bone mineral density

| Comparison | Within-study bias | Reporting bias | Indirectness | Imprecision | Heterogeneity | Incoherence | Confidence rating |
| --- | --- | --- | --- | --- | --- | --- | --- |
| GL+WMT:WMT | Some concerns | Low risk | No concerns | Major concerns | No concerns | Major concerns | Very low |
| GSK+WMT:WMT | High risk | Low risk | No concerns | No concerns | Major concerns | Major concerns | Low |
| JTG+WMT:WMT | Some concerns | Low risk | No concerns | Major concerns | No concerns | Major concerns | Very low |
| LWDHW+WMT:WMT | Some concerns | Low risk | No concerns | Major concerns | No concerns | Major concerns | Very low |
| TMK+WMT:WMT | High risk | Low risk | No concerns | Major concerns | No concerns | Major concerns | Very low |
| WMT:XLGB+WMT | Some concerns | Low risk | No concerns | No concerns | Major concerns | Major concerns | Low |
| GL+WMT:GSK+WMT | Some concerns | Low risk | No concerns | Major concerns | No concerns | Major concerns | Very low |
| GL+WMT:JTG+WMT | Some concerns | Low risk | No concerns | Major concerns | No concerns | Major concerns | Very low |
| GL+WMT:LWDHW+WMT | Some concerns | Low risk | No concerns | Major concerns | No concerns | Major concerns | Very low |
| GL+WMT:TMK+WMT | Some concerns | Low risk | No concerns | Major concerns | No concerns | Major concerns | Very low |
| GL+WMT:XLGB+WMT | Some concerns | Low risk | No concerns | Major concerns | No concerns | Major concerns | Very low |
| GSK+WMT:JTG+WMT | Some concerns | Low risk | No concerns | Major concerns | No concerns | Major concerns | Very low |
| GSK+WMT:LWDHW+WMT | High risk | Low risk | No concerns | Major concerns | No concerns | Major concerns | Very low |
| GSK+WMT:TMK+WMT | High risk | Low risk | No concerns | Major concerns | No concerns | Major concerns | Very low |
| GSK+WMT:XLGB+WMT | Some concerns | Low risk | No concerns | Major concerns | No concerns | Major concerns | Very low |
| JTG+WMT:LWDHW+WMT | Some concerns | Low risk | No concerns | Major concerns | No concerns | Major concerns | Very low |
| JTG+WMT:TMK+WMT | Some concerns | Low risk | No concerns | Major concerns | No concerns | Major concerns | Very low |
| JTG+WMT:XLGB+WMT | Some concerns | Low risk | No concerns | Major concerns | No concerns | Major concerns | Very low |
| LWDHW+WMT:TMK+WMT | High risk | Low risk | No concerns | Major concerns | No concerns | Major concerns | Very low |
| LWDHW+WMT:XLGB+WMT | Some concerns | Low risk | No concerns | Major concerns | No concerns | Major concerns | Very low |
| TMK+WMT:XLGB+WMT | Some concerns | Low risk | No concerns | Major concerns | No concerns | Major concerns | Very low |

**Table S8.3:** CINeMA Results of Femoral greater trochanter bone mineral density

| Comparison | Within-study bias | Reporting bias | Indirectness | Imprecision | Heterogeneity | Incoherence | Confidence rating |
| --- | --- | --- | --- | --- | --- | --- | --- |
| GSK+WMT:WMT | Major concerns | Low risk | No concerns | Major concerns | No concerns | Major concerns | Very low |
| JTG+WMT:WMT | Some concerns | Low risk | No concerns | No concerns | Major concerns | Major concerns | Low |
| WMT:XLGB+WMT | Some concerns | Low risk | No concerns | Major concerns | No concerns | Major concerns | Very low |
| GSK+WMT:JTG+WMT | Some concerns | Low risk | No concerns | Major concerns | No concerns | Major concerns | Very low |
| GSK+WMT:XLGB+WMT | Some concerns | Low risk | No concerns | Major concerns | No concerns | Major concerns | Very low |
| JTG+WMT:XLGB+WMT | Some concerns | Low risk | No concerns | Major concerns | No concerns | Major concerns | Very low |

**Table S8.4:** CINeMA Results of Ward’s triangle bone mineral density

| Comparison | Within-study bias | Reporting bias | Indirectness | Imprecision | Heterogeneity | Incoherence | Confidence rating |
| --- | --- | --- | --- | --- | --- | --- | --- |
| GSK+WMT:WMT | Major concerns | Low risk | No concerns | No concerns | Major concerns | Major concerns | Low |
| JTG+WMT:WMT | Some concerns | Low risk | No concerns | No concerns | Major concerns | Major concerns | Low |
| LWDHW+WMT:WMT | Some concerns | Low risk | No concerns | No concerns | Major concerns | Major concerns | Low |
| WMT:XLGB+WMT | Some concerns | Low risk | No concerns | No concerns | Major concerns | Major concerns | Low |
| GSK+WMT:JTG+WMT | Some concerns | Low risk | No concerns | Major concerns | No concerns | Major concerns | Very low |
| GSK+WMT:LWDHW+WMT | Major concerns | Low risk | No concerns | Major concerns | No concerns | Major concerns | Very low |
| GSK+WMT:XLGB+WMT | Some concerns | Low risk | No concerns | Major concerns | No concerns | Major concerns | Very low |
| JTG+WMT:LWDHW+WMT | Some concerns | Low risk | No concerns | Major concerns | No concerns | Major concerns | Very low |
| JTG+WMT:XLGB+WMT | Some concerns | Low risk | No concerns | Major concerns | No concerns | Major concerns | Very low |
| LWDHW+WMT:XLGB+WMT | Some concerns | Low risk | No concerns | Major concerns | No concerns | Major concerns | Very low |

**Table S8.5:** CINeMA Results of Osteocalcin

| Comparison | Within-study bias | Reporting bias | Indirectness | Imprecision | Heterogeneity | Incoherence | Confidence rating |
| --- | --- | --- | --- | --- | --- | --- | --- |
| GL+WMT:WMT | Some concerns | Low risk | No concerns | No concerns | Major concerns | Major concerns | Low |
| GSK+WMT:WMT | Major concerns | Low risk | No concerns | No concerns | Major concerns | Major concerns | Low |
| JTG+WMT:WMT | Some concerns | Low risk | No concerns | No concerns | No concerns | Major concerns | Moderate |
| LWDHW+WMT:WMT | Some concerns | Low risk | No concerns | No concerns | Major concerns | Major concerns | Low |
| TMK+WMT:WMT | Some concerns | Low risk | No concerns | Major concerns | No concerns | Major concerns | Very low |
| WMT:XLGB+WMT | Some concerns | Low risk | No concerns | No concerns | No concerns | Major concerns | Moderate |
| GL+WMT:GSK+WMT | Some concerns | Low risk | No concerns | Major concerns | No concerns | Major concerns | Very low |
| GL+WMT:JTG+WMT | Some concerns | Low risk | No concerns | No concerns | Major concerns | Major concerns | Low |
| GL+WMT:LWDHW+WMT | Some concerns | Low risk | No concerns | Major concerns | No concerns | Major concerns | Very low |
| GL+WMT:TMK+WMT | Some concerns | Low risk | No concerns | Major concerns | No concerns | Major concerns | Very low |
| GL+WMT:XLGB+WMT | Some concerns | Low risk | No concerns | No concerns | No concerns | Major concerns | Moderate |
| GSK+WMT:JTG+WMT | Some concerns | Low risk | No concerns | Major concerns | No concerns | Major concerns | Very low |
| GSK+WMT:LWDHW+WMT | Major concerns | Low risk | No concerns | Major concerns | No concerns | Major concerns | Very low |
| GSK+WMT:TMK+WMT | Major concerns | Low risk | No concerns | Major concerns | No concerns | Major concerns | Very low |
| GSK+WMT:XLGB+WMT | Some concerns | Low risk | No concerns | No concerns | No concerns | Major concerns | Moderate |
| JTG+WMT:LWDHW+WMT | Some concerns | Low risk | No concerns | No concerns | Major concerns | Major concerns | Low |
| JTG+WMT:TMK+WMT | Some concerns | Low risk | No concerns | No concerns | Major concerns | Major concerns | Low |
| JTG+WMT:XLGB+WMT | Some concerns | Low risk | No concerns | No concerns | No concerns | Major concerns | Moderate |
| LWDHW+WMT:TMK+WMT | Some concerns | Low risk | No concerns | Major concerns | No concerns | Major concerns | Very low |
| LWDHW+WMT:XLGB+WMT | Some concerns | Low risk | No concerns | No concerns | No concerns | Major concerns | Moderate |
| TMK+WMT:XLGB+WMT | Some concerns | Low risk | No concerns | No concerns | No concerns | Major concerns | Moderate |

**Table S8.6:** CINeMA Results of ALP

| Comparison | Within-study bias | Reporting bias | Indirectness | Imprecision | Heterogeneity | Incoherence | Confidence rating |
| --- | --- | --- | --- | --- | --- | --- | --- |
| GL+WMT:WMT | Some concerns | Low risk | No concerns | Major concerns | No concerns | Major concerns | Very low |
| GSK+WMT:WMT | Some concerns | Low risk | No concerns | Major concerns | No concerns | Major concerns | Very low |
| JTG+WMT:WMT | Some concerns | Low risk | No concerns | Major concerns | No concerns | Major concerns | Very low |
| LWDHW+WMT:WMT | Some concerns | Low risk | No concerns | No concerns | No concerns | Major concerns | Moderate |
| TMK+WMT:WMT | Some concerns | Low risk | No concerns | Major concerns | No concerns | Major concerns | Very low |
| GL+WMT:GSK+WMT | Some concerns | Low risk | No concerns | Major concerns | No concerns | Major concerns | Very low |
| GL+WMT:JTG+WMT | Some concerns | Low risk | No concerns | Major concerns | No concerns | Major concerns | Very low |
| GL+WMT:LWDHW+WMT | Some concerns | Low risk | No concerns | No concerns | No concerns | Major concerns | Moderate |
| GL+WMT:TMK+WMT | Some concerns | Low risk | No concerns | Major concerns | No concerns | Major concerns | Very low |
| GSK+WMT:JTG+WMT | Some concerns | Low risk | No concerns | Major concerns | No concerns | Major concerns | Very low |
| GSK+WMT:LWDHW+WMT | Some concerns | Low risk | No concerns | No concerns | No concerns | Major concerns | Moderate |
| GSK+WMT:TMK+WMT | Some concerns | Low risk | No concerns | Major concerns | No concerns | Major concerns | Very low |
| JTG+WMT:LWDHW+WMT | Some concerns | Low risk | No concerns | No concerns | No concerns | Major concerns | Moderate |
| JTG+WMT:TMK+WMT | Some concerns | Low risk | No concerns | Major concerns | No concerns | Major concerns | Very low |
| LWDHW+WMT:TMK+WMT | Some concerns | Low risk | No concerns | No concerns | No concerns | Major concerns | Moderate |

**Table S8.7:** CINeMA Results of Ca

| Comparison | Within-study bias | Reporting bias | Indirectness | Imprecision | Heterogeneity | Incoherence | Confidence rating |
| --- | --- | --- | --- | --- | --- | --- | --- |
| GSK+WMT:WMT | Major concerns | Low risk | No concerns | No concerns | Major concerns | Major concerns | Low |
| JTG+WMT:WMT | Some concerns | Low risk | No concerns | Major concerns | No concerns | Major concerns | Very low |
| LWDHW+WMT:WMT | Some concerns | Low risk | No concerns | Major concerns | No concerns | Major concerns | Very low |
| TMK+WMT:WMT | Some concerns | Low risk | No concerns | Major concerns | No concerns | Major concerns | Very low |
| WMT:XLGB+WMT | Some concerns | Low risk | No concerns | No concerns | Major concerns | Major concerns | Low |
| GSK+WMT:JTG+WMT | Some concerns | Low risk | No concerns | Major concerns | No concerns | Major concerns | Very low |
| GSK+WMT:LWDHW+WMT | Some concerns | Low risk | No concerns | Major concerns | No concerns | Major concerns | Very low |
| GSK+WMT:TMK+WMT | Some concerns | Low risk | No concerns | Major concerns | No concerns | Major concerns | Very low |
| GSK+WMT:XLGB+WMT | Some concerns | Low risk | No concerns | Major concerns | No concerns | Major concerns | Very low |
| JTG+WMT:LWDHW+WMT | Some concerns | Low risk | No concerns | Major concerns | No concerns | Major concerns | Very low |
| JTG+WMT:TMK+WMT | Some concerns | Low risk | No concerns | Major concerns | No concerns | Major concerns | Very low |
| JTG+WMT:XLGB+WMT | Some concerns | Low risk | No concerns | No concerns | Major concerns | Major concerns | Low |
| LWDHW+WMT:TMK+WMT | Some concerns | Low risk | No concerns | Major concerns | No concerns | Major concerns | Very low |
| LWDHW+WMT:XLGB+WMT | Some concerns | Low risk | No concerns | No concerns | Major concerns | Major concerns | Low |
| TMK+WMT:XLGB+WMT | Some concerns | Low risk | No concerns | Major concerns | No concerns | Major concerns | Very low |

**Table S8.8:** CINeMA Results of P

| Comparison | Within-study bias | Reporting bias | Indirectness | Imprecision | Heterogeneity | Incoherence | Confidence rating |
| --- | --- | --- | --- | --- | --- | --- | --- |
| GSK+WMT:WMT | Major concerns | Low risk | No concerns | Major concerns | No concerns | No concerns | Very low |
| JTG+WMT:WMT | Some concerns | Low risk | No concerns | Major concerns | No concerns | No concerns | Low |
| TMK+WMT:WMT | Some concerns | Low risk | No concerns | Major concerns | No concerns | No concerns | Low |
| WMT:XLGB+WMT | Some concerns | Low risk | No concerns | No concerns | No concerns | No concerns | High |
| GSK+WMT:JTG+WMT | Major concerns | Low risk | No concerns | Major concerns | No concerns | No concerns | Very low |
| GSK+WMT:TMK+WMT | Some concerns | Low risk | No concerns | Major concerns | No concerns | No concerns | Low |
| GSK+WMT:XLGB+WMT | Some concerns | Low risk | No concerns | No concerns | No concerns | No concerns | High |
| JTG+WMT:TMK+WMT | Some concerns | Low risk | No concerns | Major concerns | No concerns | No concerns | Low |
| JTG+WMT:XLGB+WMT | Some concerns | Low risk | No concerns | No concerns | No concerns | No concerns | High |
| TMK+WMT:XLGB+WMT | Some concerns | Low risk | No concerns | Major concerns | No concerns | No concerns | Low |

**Table S8.9:** CINeMA Results of FBG

| Comparison | Within-study bias | Reporting bias | Indirectness | Imprecision | Heterogeneity | Incoherence | Confidence rating |
| --- | --- | --- | --- | --- | --- | --- | --- |
| GSK+WMT:WMT | Some concerns | Low risk | No concerns | Major concerns | No concerns | Major concerns | Very low |
| JTG+WMT:WMT | Some concerns | Low risk | No concerns | Major concerns | No concerns | Major concerns | Very low |
| LWDHW+WMT:WMT | Some concerns | Low risk | No concerns | No concerns | Major concerns | Major concerns | Low |
| TMK+WMT:WMT | Major concerns | Low risk | No concerns | Major concerns | No concerns | Major concerns | Very low |
| GSK+WMT:JTG+WMT | Some concerns | Low risk | No concerns | Major concerns | No concerns | Major concerns | Very low |
| GSK+WMT:LWDHW+WMT | Some concerns | Low risk | No concerns | Major concerns | No concerns | Major concerns | Very low |
| GSK+WMT:TMK+WMT | Some concerns | Low risk | No concerns | Major concerns | No concerns | Major concerns | Very low |
| JTG+WMT:LWDHW+WMT | Some concerns | Low risk | No concerns | Major concerns | No concerns | Major concerns | Very low |
| JTG+WMT:TMK+WMT | Some concerns | Low risk | No concerns | Major concerns | No concerns | Major concerns | Very low |
| LWDHW+WMT:TMK+WMT | Major concerns | Low risk | No concerns | Major concerns | No concerns | Major concerns | Very low |

**Table S8.10:** CINeMA Results of Total effective rate

| Comparison | Within-study bias | Reporting bias | Indirectness | Imprecision | Heterogeneity | Incoherence | Confidence rating |
| --- | --- | --- | --- | --- | --- | --- | --- |
| GL+WMT:WMT | Some concerns | Low risk | No concerns | No concerns | No concerns | No concerns | High |
| GSK+WMT:WMT | Some concerns | Low risk | No concerns | No concerns | No concerns | No concerns | High |
| JTG+WMT:WMT | Some concerns | Low risk | No concerns | No concerns | No concerns | No concerns | High |
| LWDHW+WMT:WMT | Some concerns | Low risk | No concerns | No concerns | No concerns | No concerns | High |
| TMK+WMT:WMT | Major concerns | Low risk | No concerns | No concerns | No concerns | No concerns | Moderate |
| WMT:XLGB+WMT | Some concerns | Low risk | No concerns | No concerns | No concerns | No concerns | High |
| GL+WMT:GSK+WMT | Some concerns | Low risk | No concerns | Major concerns | No concerns | No concerns | Low |
| GL+WMT:JTG+WMT | Some concerns | Low risk | No concerns | Major concerns | No concerns | No concerns | Low |
| GL+WMT:LWDHW+WMT | Some concerns | Low risk | No concerns | Major concerns | No concerns | No concerns | Low |
| GL+WMT:TMK+WMT | Some concerns | Low risk | No concerns | Major concerns | No concerns | No concerns | Low |
| GL+WMT:XLGB+WMT | Some concerns | Low risk | No concerns | Major concerns | No concerns | No concerns | Low |
| GSK+WMT:JTG+WMT | Some concerns | Low risk | No concerns | No concerns | No concerns | No concerns | High |
| GSK+WMT:LWDHW+WMT | Some concerns | Low risk | No concerns | Major concerns | No concerns | No concerns | Low |
| GSK+WMT:TMK+WMT | Some concerns | Low risk | No concerns | Major concerns | No concerns | No concerns | Low |
| GSK+WMT:XLGB+WMT | Some concerns | Low risk | No concerns | No concerns | No concerns | No concerns | High |
| JTG+WMT:LWDHW+WMT | Some concerns | Low risk | No concerns | Major concerns | No concerns | No concerns | Low |
| JTG+WMT:TMK+WMT | Some concerns | Low risk | No concerns | Major concerns | No concerns | No concerns | Low |
| JTG+WMT:XLGB+WMT | Some concerns | Low risk | No concerns | Major concerns | No concerns | No concerns | Low |
| LWDHW+WMT:TMK+WMT | Some concerns | Low risk | No concerns | Major concerns | No concerns | No concerns | Low |
| LWDHW+WMT:XLGB+WMT | Some concerns | Low risk | No concerns | Major concerns | No concerns | No concerns | Low |
| TMK+WMT:XLGB+WMT | Some concerns | Low risk | No concerns | Major concerns | No concerns | No concerns | Low |

**Table S8.11:** CINeMA Results of Adverse events

| Comparison | Within-study bias | Reporting bias | Indirectness | Imprecision | Heterogeneity | Incoherence | Confidence rating |
| --- | --- | --- | --- | --- | --- | --- | --- |
| GL+WMT:WMT | Some concerns | Low risk | No concerns | Major concerns | No concerns | No concerns | Low |
| JTG+WMT:WMT | Some concerns | Low risk | No concerns | Major concerns | No concerns | No concerns | Low |
| LWDHW+WMT:WMT | Some concerns | Low risk | No concerns | Major concerns | No concerns | No concerns | Low |
| TMK+WMT:WMT | Major concerns | Low risk | No concerns | Major concerns | No concerns | No concerns | Very low |
| WMT:XLGB+WMT | Some concerns | Low risk | No concerns | Major concerns | No concerns | No concerns | Low |
| GL+WMT:JTG+WMT | Some concerns | Low risk | No concerns | Major concerns | No concerns | No concerns | Low |
| GL+WMT:LWDHW+WMT | Some concerns | Low risk | No concerns | Major concerns | No concerns | No concerns | Low |
| GL+WMT:TMK+WMT | Some concerns | Low risk | No concerns | Major concerns | No concerns | No concerns | Low |
| GL+WMT:XLGB+WMT | Some concerns | Low risk | No concerns | Major concerns | No concerns | No concerns | Low |
| JTG+WMT:LWDHW+WMT | Some concerns | Low risk | No concerns | Major concerns | No concerns | No concerns | Low |
| JTG+WMT:TMK+WMT | Major concerns | Low risk | No concerns | Major concerns | No concerns | No concerns | Very low |
| JTG+WMT:XLGB+WMT | Some concerns | Low risk | No concerns | Major concerns | No concerns | No concerns | Low |
| LWDHW+WMT:TMK+WMT | Some concerns | Low risk | No concerns | Major concerns | No concerns | No concerns | Low |
| LWDHW+WMT:XLGB+WMT | Some concerns | Low risk | No concerns | Major concerns | No concerns | No concerns | Low |
| TMK+WMT:XLGB+WMT | Some concerns | Low risk | No concerns | Major concerns | No concerns | No concerns | Low |

**Appendix 9: Funnel plots**

**Figure S9.1**: Funnel plot of Lumbar spine bone mineral density

**Figure S9.2**: Funnel plot of Femoral neck bone mineral density

**Figure S9.3**: Funnel plot of Femoral greater trochanter bone mineral density

**Figure S9.4**: Funnel plot of Ward’s triangle bone mineral density

**Figure S9.5**: Funnel plot of Osteocalcin

**Figure S9.6**: Funnel plot of ALP

**Figure S9.7**: Funnel plot of Ca

**Figure S9.8**: Funnel plot of P

**Figure S9.9**: Funnel plot of FBG

**Figure S9.10**: Funnel plot of Total effective rate

**Figure S9.11**: Funnel plot of adverse events
